# Differential neurovirulence of Usutu virus lineages in mice and neuronal cells

**DOI:** 10.1186/s12974-020-02060-4

**Published:** 2021-01-06

**Authors:** Marion Clé, Orianne Constant, Jonathan Barthelemy, Caroline Desmetz, Marie France Martin, Lina Lapeyre, Daniel Cadar, Giovanni Savini, Liana Teodori, Federica Monaco, Jonas Schmidt-Chanasit, Juan-Carlos Saiz, Gaëlle Gonzales, Sylvie Lecollinet, Cécile Beck, Fabien Gosselet, Philippe Van de Perre, Vincent Foulongne, Sara Salinas, Yannick Simonin

**Affiliations:** 1 Pathogenesis and Control of Chronic Infections, Université de Montpellier, INSERM, EFS, Montpellier, France; 2 BioCommunication en CardioMétabolique (BC2M), Montpellier University, Montpellier, France; 3 Université de Montpellier, CNRS, Viral Trafficking, Restriction and Innate Signaling, Montpellier, France; 4 Bernhard Nocht Institute for Tropical Medicine, WHO Collaborating Centre for Arbovirus and Haemorrhagic Fever Reference and Research, 20359 Hamburg, Germany; 5 OIE Reference Centre for West Nile Disease, Istituto Zooprofilattico Sperimentale “G. Caporale”, 46100 Teramo, Italy; 6 Faculty of Mathematics, Informatics and Natural Sciences, Universität Hamburg, 20148 Hamburg, Germany; 7 Department of Biotechnology, INIA, Madrid, Spain; 8 UPE, Anses Animal Health Laboratory, UMR1161 Virology, INRA, Anses, ENVA, Maisons-Alfort, France; 9 Blood-Brain Barrier Laboratory (BBB Lab), University of Artois, UR2465, F-62300 Lens, France; 10 Centre Hospitalier Universitaire de Montpellier, Montpellier, France

**Keywords:** Usutu virus, Arbovirus, Flavivirus, Neurotropism, Central nervous system

## Abstract

**Background:**

Usutu virus (USUV) is an emerging neurotropic arthropod-borne virus recently involved in massive die offs of wild birds predominantly reported in Europe. Although primarily asymptomatic or presenting mild clinical signs, humans infected by USUV can develop neuroinvasive pathologies (including encephalitis and meningoencephalitis). Similar to other flaviviruses, such as West Nile virus, USUV is capable of reaching the central nervous system. However, the neuropathogenesis of USUV is still poorly understood, and the virulence of the specific USUV lineages is currently unknown. One of the major complexities of the study of USUV pathogenesis is the presence of a great diversity of lineages circulating at the same time and in the same location.

**Methods:**

The aim of this work was to determine the neurovirulence of isolates from the six main lineages circulating in Europe using mouse model and several neuronal cell lines (neurons, microglia, pericytes, brain endothelial cells, astrocytes, and in vitro Blood-Brain Barrier model).

**Results:**

Our results indicate that all strains are neurotropic but have different virulence profiles. The Europe 2 strain, previously described as being involved in several clinical cases, induced the shortest survival time and highest mortality in vivo and appeared to be more virulent and persistent in microglial, astrocytes, and brain endothelial cells, while also inducing an atypical cytopathic effect. Moreover, an amino acid substitution (D3425E) was specifically identified in the RNA-dependent RNA polymerase domain of the NS5 protein of this lineage.

**Conclusions:**

Altogether, these data show a broad neurotropism for USUV in the central nervous system with lineage-dependent virulence. Our results will help to better understand the biological and epidemiological diversity of USUV infection.

## Background

Usutu virus (USUV) is an arbovirus of the genus *Flavivirus* from the *Flaviviridae* family. It belongs to the Japanese encephalitis virus (JEV) antigenic complex isolated for the first time in 1959 from *Culex neavei* (trapped in Swaziland) after intracerebral inoculation of newborn mice in Southern Africa [1–4]. Over recent years, this emerging virus has dispersed out of Africa mainly into Europe [5]. It was presumably carried by migratory birds and their associated mosquitoes, such as *Culex pipiens* [6–9]. Similar to other flaviviruses, USUV is an icosahedral-enveloped virus with a single-stranded positive sense RNA of 11 kb, with a 5′ cap structure and one open reading frame (ORF) encoding a polyprotein of 3434 amino acids. The USUV polyprotein is post-translationally processed by cellular and viral proteases into three structural proteins: capsid (C), pre-membrane/membrane (prM/M), and envelope (E), and seven nonstructural proteins (NS1, NS2A, NS2B, NS3, NS4A, NS4B, and NS5) [10]. Its natural life cycle mainly involves birds (like *Strigiformes* or *Passeriformes*) and mosquitoes (mainly *Culex*) [11].

USUV infection of birds is frequently characterized by encephalitis, myocardial degeneration, and necrosis of the brain, liver, and spleen [6, 12]. Humans and others mammals, such as horses, rodents, dogs, and wild boars, are considered accidental hosts [13–17]. The first two cases of USUV infection in humans were described in Africa, precisely in Central African Republic (1981) and Burkina Faso (2004), with mild symptoms such as fever and rash [18]. In Europe, USUV infection was reported for the first time in 2009 in two patients in Italy [19–21]*.* Since then, several studies have reported human infections in Italy associated with neurological damages [22–27]*.* In 2013 and 2018, six patients with neuroinvasive symptoms (meningitis and meningoencephalitis) were diagnosed in Croatia, and a patient with aseptic meningitis in Hungary [28–30]. In 2016, USUV infection was reported in France in a patient with idiopathic facial paralysis [31]. In addition, USUV was detected in asymptomatic human blood donors in Austria, Germany, and the Netherlands [32–36]. To date, 100 cases of acute human infection have been described, mainly in Europe including patients with neurological symptoms (Table 1). Major USUV epizootics affecting avifauna, associated with a large epidemic of West Nile virus (WNV), were demonstrated in Europe in 2016 and in 2018 [37].

**Table 1:** Description of human cases of USUV infection worldwide

Country	Year	Number	Sample	Clinical signs	Studied population	Identified strains	Ref
CAR	1981	1	Blood	Eruptive fever	Clinical case	Africa 3	[18]
**Burkina Faso**	2004	1	Blood	Fever and jaundice	Clinical case	?	[18]
**Italy**	2009	1	CSF	Meningoencephalitis	Clinical case	Europe 1	[19]
	2009	1	Blood	Encephalitis	Clinical case	Europe 2	[20, 21]
	2008–2009	3/44	CSF	Meningoencephalitis	Meningoencephalitis patients	?	[22]
	2008–2011	8/306 + 2/609	CSF / Blood	Meningoencephalitis / Healthy	Meningoencephalitis patients (CSF). various healthy and sick subjects (serum)	?	[23]
	2018	1/44	Blood	Healthy	Blood donors	Europe 1	[24]
	2016–2018	25/73964	Blood	Healthy	Blood donors	?	[25]
	2017–2018	9	Blood	Healthy	Blood donors	Europe 2. Europe 3 and Europe 4	[26]
	2017–2018	8/1967	Blood / Urine	Neuroinvasive diseases / Fever / Arthralgia / Myalgia	Patients with suspected arboviral infection	Europe 2	[27]
**Croatia**	2013	3/95	Blood	Meningoencephalitis	Meningoencephalitis patients	?	[29]
	2018	3/178	Blood / Urine	Neuroinvasive diseases	Neuroinvasive cohort	Europe 2	[30]
**Germany**	2016	1	Blood	Healthy	Blood donors	Europe 3	[34]
**Netherlands**	2018	7/12040	Blood	Healthy	Blood donors	Europe 3	[36]
**France**	2016	1/166	CSF	Idiopathic facial paralysis	Patients with infection and/or neurological signs	Africa 2	[31]
**Austria**	2017	6/12047	Blood	Healthy	Blood donors	Europe 2	[32]
	2018	18/31598	Blood	Healthy / Rash	Blood donors	Europe 2 and Africa 3	[33]
**Hungary**	2018	1	Blood	Aseptic meningitis	Clinical case	Europe 2	[28]

This table summarizes acute human cases of USUV infection described in the literature and the viral strains involved

*CAR* Central African Republic

Isolates of USUV are currently classified into eight different genetic lineages which are divided in two major African or European groups: Africa (AF) 1, 2, and 3 and Europe (EU) 1, 2, 3, 4, and 5 [38, 39]. Although the great USUV evolutionary diversity in Europe appears to have emerged in the last decade, phylogeny analyses suggest relatively long-term circulation of USUV in Europe. As shown in Table 1, human cases of USUV infection are associated with different lineages. However, it is interesting to note that the majority of patients developing neurological symptoms are related to the EU2 strain, which is actively circulating in several European countries including Italy. It has been reported in the literature that among 24 patients presenting neurological symptoms, 13 cases were infected with EU2 [20, 27, 28, 30], one with AF2 [31], and one with EU1 [19], while for the other cases the strains involved were not characterized. USUV isolates have also been identified in asymptomatic infected patients or infected patients with mild clinical signs, such as AF3 [18, 33], EU1 [24], EU2 [26, 32, 33], EU3 [26, 34, 36], and EU4 [26]. Complete genome sequencing and phylogenetic analyses of African and European lineages have shown genetic diversity among all USUV strains analyzed [40]. The pathogenicity of USUV strains currently circulating in Europe still remains to be investigated in order to understand the epidemiology and evolution of USUV. It has been well described that the neuroinvasive potential of many flaviviruses is strain-dependent [41, 42]*.* This is the case for WNV which shares a lot of similarities with USUV [43]. However, little is known with respect to the neurovirulence of USUV lineages, and it could be hypothesized that the lineages currently circulating in Europe differ according to their virulence profiles.

The aim of this present study was to characterize the viral neurotropism and neuropathogenicity of six USUV strains (EU1, EU2, EU3, EU5, AF2, and AF3 lineages) currently circulating in Europe, using a suckling immunocompetent mouse model and both murine and human brain cells. Despite the fact that all USUV strains were neurotropic and lethal in vivo, we observed different rates of replication, cytokine induction, and mortality between the different lineages. The EU2 isolate was the most virulent, with 100% mortality in vivo and induction of symptoms comparable to epileptic seizures. These symptoms were not observed among the other strains. In vitro, we found that all isolates infected cerebral cells types, such as murine neurons and microglia, human pericytes, brain endothelial cells, and astrocytes. However, in vitro infection showed that USUV isolates gave rise to different growth dynamics. Interestingly, the EU2 lineage exhibited the highest cell infection level, with both higher and more persistent replication rates, while also inducing an atypical cytopathic effect (CPE) characterized by dark cell clusters that detached from the cell mat.

## Materials and methods

### USUV strains and cellular infection

Europe 1 strain (Vienna 2001-blackbird) was from INIA Madrid (Spain); Europe 2 (TE20421/Italy/2017 and TE18982/Italy/2017) was provided from Istituto Zooprofilattico Sperimentale, Emilia Romagna (Italy); Europe 3 (USUV-HautRhin7315/France/2015), Africa 2 (Rhône 2705/France/2015), and Africa 3 (USUV-HauteVienne4997/France/2018) strains were from ANSES (National Agency for Food, Environmental and Occupational Health Safety, France); and the Europe 5 (BNI-507/2016/Germany) strain was coming from Bernhard Nocht Institute for Tropical Medicine, Hamburg (Germany) (Table 2). The virus lineages were amplified no more than four times on Vero cells (ATCC CCL-81). Viral stocks were prepared by infecting 80% confluent Vero cells, and cellular supernatants were collected 5 to 7 days after infection. Viral titers were determined by TCID50/ml, which were calculated using the Spearman-Kärber method [44]. For infections, cells at sub confluence were rinsed once with PBS, and USUV was added in a small volume of medium for 2 h at 37 °C with constant agitation prior to removing the inoculum.

**Table 2:** Country, year of isolation, host source, and passage history of the USUV strains used in the study

Lineage	Strains Name	Country of isolation	Year of islolation	Isolation source	Number of passages	Ref/accession number
Europe 1	Vienna-2001-blackbird	Austria	2001	Avian	4	AY453411.1
Europe 2	TE20421/Italy/2017	Italy	2017	Avian	3	TE20421
Europe 3	USUV-HautRhin7315	France	2015	Avian	4	KX601690
Europe 5	BNI507/2016/Germany	Germany	2016	Avian	3	KY113091
Africa 2	Rhône 2705	France	2015	Avian	3	KX601692
Africa 3	USUV-HauteVienne4977	France	2018	Avian	4	AF4997 (MT863562)

### Mouse experiments

Six-day-old neonatal Swiss mice (Janvier Laboratories, Saint-Berthevin Cedex, France) were inoculated intraperitoneally with 10^3^ TCID50/mice of USUV (*n* = 10 to 12 mice per group). Cervical dislocation was used to euthanize mice at the end of the experiment or if mice presented general deterioration in their condition. Tissues were snap frozen in liquid nitrogen for the determination of the viral burden or fixed in 4% paraformaldehyde (PFA) and cut using a microtome (3-μm sections) at the RHEM facilities (Montpellier) for histological analysis.

### Histology and immunostaining

Briefly, samples were collected and fixed for 24 h in neutral buffered formalin 10% and embedded in paraffin. Tissues were cut into 3-μm-thick sections, mounted on slides, then dried at 37 °C overnight, and stained as previously described [45]. The slides were incubated at 37 °C for 60 min with a rat anti-CD45 antibody (14-0451, Bioscience) or a rabbit anti-Cleaved caspase 3 antibody (9671, Cell signaling). For viral antigen immunostaining, the slides were incubated at 37 °C for 32 min with a mouse anti-flavivirus group antigen monoclonal antibody (Millipore, MAB10216). A scanner (Hamamatsu NanoZoomer 2.0-HT) was used to analyze the slides. Staining cells were quantified with the QuPath bioimage analysis software.

### Viral quantification for in vivo experiments

Organs were crushed with zirconia beads (Fastprep apparatus, MP Biomedicals) in 500 μL PBS before RNA extraction (RNA RNeasy Mini-Kit, Qiagen). Urine and blood RNA were extracted with the EZ1 DSP virus kit (Qiagen), and viral RNA levels were quantified by a one-step quantitative reverse transcriptase PCR assay (RT-qPCR) (Light Cycler 480, Roche) as previously described [46]. Viral load was reported as TCID50 equivalents per gram using a standard curve produced using serial 10-fold dilutions of USUV with known viral titers.

### Cells

Vero cells (ATCC CCL-81) and immortalized murine microglial cell line BV-2 (generated by Dr. Elisabetta Blasi [47]) were cultured in DMEM medium containing 10% fetal bovine serum (FBS). C6/36 cells (ATCC CRL-1660) were grown in RPMI medium with 10% of FBS. Human pericytes and astrocytes cells (catalog #1200 and #1830, ScienCell^TM^ ) were cultured on poly-l-lysine–coated plates. Human endothelial cells were purchased from ScienCell^TM^ and cultured on fibronectin-coated plates. Mouse hippocampal neurons (E18) were obtained and plated using standard procedures as previously described [44].

### Immunofluorescence assays

Cells were fixed with 4% PFA and permeabilized for 5 min at room temperature with 0.1% Triton X-100. After a blocking step for 30 min to 1 h at RT with 2% bovine serum albumin (BSA), an incubation step with primary and secondary antibodies was performed. Cells were then fixed and incubated during 5 min with Hoechst (Sigma) and mounted with fluorescent mounting medium (Prolongold, Thermo Fisher). Samples were imaged by the Zeiss SP85 confocal microscope. Infected cells were quantified by counting 10 fields per conditions per experiments (≥ 1000 cells, *n* = 3).

### ELISA (enzyme-linked immunosorbent assay)

At various days post-infection, ELISA assays for human CCL5, IL6, and CXCL10 were performed in mock- and USUV-infected supernatants from microglia cells and astrocytes cells (R&D systems). Data were analyzed on a spectrophotometer (Thermo Fisher Scientific). Absorbance was measured at 450 nm. Mean concentrations (pg/mL) of cytokines were all superior to the detection limits, defined as the mean background value plus two standard deviations (stddev).

### RT-qPCR assays

USUV- or mock-infected cells (microglia and astrocytes) or mouse brain homogenates were harvested in RLT buffer (Qiagen), and RNA was extracted using RNeasy Mini-Kit (Qiagen). cDNA was synthesized (Omniscript reverse transcriptase, Life Technologies), and then analysis was done with a LC480 real-time PCR instrument (Roche). HPRT gene was used to normalize gene expression.

### Brdu test

Cell proliferation was evaluated by enclosing 5000 cells in 96-well plates. Astrocytes cells were infected with six USUV lineages at a multiplicity of infection (MOI) 0.1. At 7 dpi, a bromodeoxyuridine ELISA test was performed (Calbiochem BrDU cell proliferation test) according to the manufacturer’s instructions. Absorbance was measured using a spectrophotometer at 450 nm (TECAN).

### MTT test

Cellular apoptosis was evaluated by enclosing 5000 cells in 96-well plates. Astrocytes cells were infected with six USUV lineages at a MOI 0.1. At 4 dpi, the test was performed according to the manufacturer’s instructions (Calbiochem, 3-(4,5-dimethyl-2-thiazolyl)-2,5-diphenyl-2H-tetrazolium Bromide, Thiazole Blue, MTT - CAS 298-93-1). Absorbance at 570 nm was measured using a plate reader.

### In vitro blood-brain barrier (BBB) model

Briefly, the in vitro human BBB models consist in CD34^+^ blood cord-derived endothelial cells (CD34^+^-EC) that have been differentiated and cultured in transwell filters (Costar) on top of bovine pericytes [48]. Medium was changed every 2 days. Lucifer Yellow (LY; 20 μM; Life Technologies) was used to test the endothelial permeability (Pe) by calculating the concentration-independent parameter, and the fluorescence was detected at 432/538 nm [48, 49]. If the barrier is impermeable (Pe ≤ to 1 × 10^−3^ cm/min), this model was infected with USUV at the MOI of 0.1. After 2 h, the inoculum was removed and 500 μL of fresh medium was added. The medium was also changed at 4, 7, or 10 dpi.

### Transendothelial electrical resistance (TEER) measurements

According to the manufacturer’s instructions, the TEER was evaluated with the Epithelial Volt/Ohm Meter EVOM2 (World Precision Instruments, Hertfordshire). Electrodes were equilibrated in media and placed in the upper and lower chambers. Background measurement of a Matrigel-coated insert (with no cells) was subtracted from the reading and the value was multiplied by the growth surface area, to determine the TEER values (Ohms*cm^2^).

### Determination of neuropathogenicity markers

Neurovirulence and neuroinvasiveness markers such as predicted N-linked glycosylation sites or the occurrence of certain amino acids described to be associated with increased virulence were determined as described previously [50]. Amino acid sequences of the polyprotein of each viral strain were aligned using MUSCLE (MUltiple Sequence Comparison by Log-Expectation, EMBL-EBI). A percent identity matrix was generated aside with the alignment (Fig. 9a). Some N-linked glycosylation sites as well as specific mutations were highlighted (Fig. 9b).

### Statistical analyses

A least three independent experiments with three replicates were done for each experiment. Student’s *t* test and the Mann-Whitney (*p < 0.05, **p < 0.01) were used to analyze unpaired data with GraphPad Prism software.

## Results

### USUV isolates are neurotropic but exhibit different virulence profiles in suckling immunocompetent mice

**Fig. 1:**
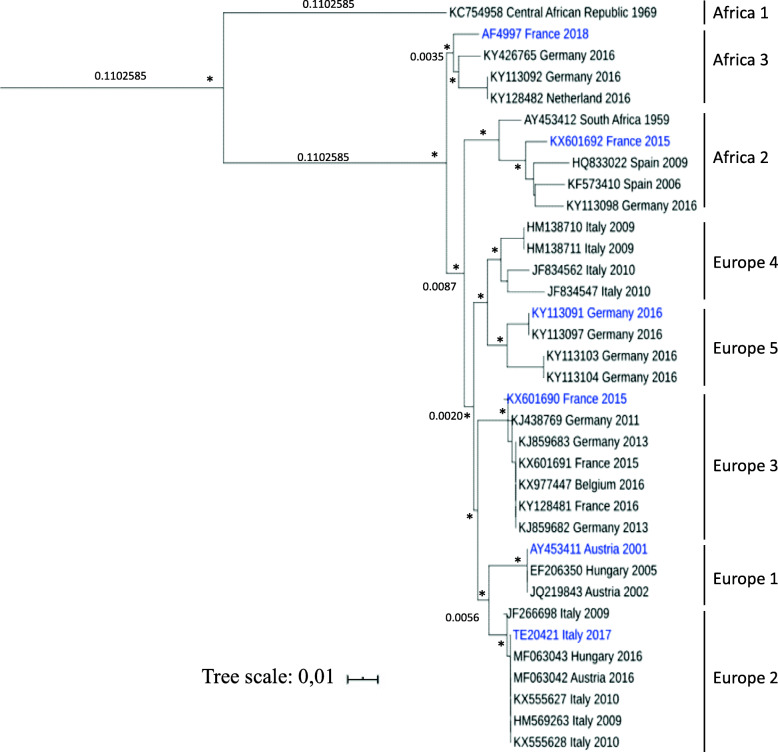
Phylogenetic analysis of Usutu virus strains. Phylogenetic tree based on the partial NS5 gene and showing positioning of the USUV isolates used in this study (in blue). USUV sequences are labeled with their respective ID or GenBank accession number, preceded by country and year of isolation. Scale bar indicates mean number of nt substitutions per site. The analysis was processed through on line phylogeny analysis (http://www.phylogeny.fr; Réseau National des Génopoles), and tree was rooted with an USUV Africa 1 sequence as the outgroup virus (GenBank accession number KC754958). The asterisks (*) at major nodes indicate an SH-like support value > 70%

It remains to be described whether all USUV strains can enter the central nervous system (CNS) and cause similar damage to the brain parenchyma or whether lesions are strain-specific. To better characterize the neurovirulence of USUV isolates, we first evaluated the susceptibility of mice to six different USUV lineages (EU1, EU2, EU3, EU5, AF2, and AF3). These isolates were at low passage numbers in order to avoid accumulation of mutations owing to extensive passaging (Table 2, Fig. 1). There is a limited number of experimental models that enable the study of flavivirus virulence because wild-type adult mice are resistant to infection by many of these viruses, including Dengue virus (DENV), USUV, and Zika virus (ZIKV) [51, 52]. To overcome this problem, it is possible to use transgenic mouse models. Mice lacking the interferon type 1 receptor (*Ifnar1*^*-/-*^) display enhanced susceptibility to infection by flaviviruses [53, 54], and immunocompetent suckling mice are susceptible to viral infection given they have not yet fully developed a functional interferon (IFN) response [51].

**Fig. 2:**
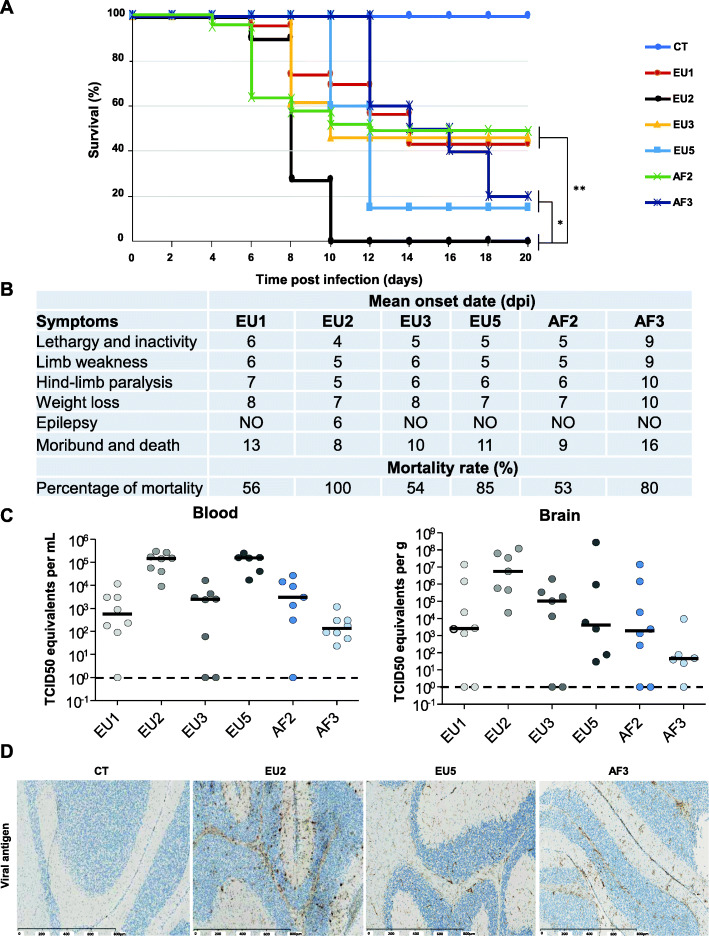
Clinical signs of USUV infection in suckling immunocompetent mice according to strains. **a** Survival rates of neonatal Swiss mice (6 days old) intraperitoneally inoculated with 10^3^ TCID50/mouse of six different USUV lineages. CT = control mice. **b** Onset date of symptoms in USUV-infected suckling mice. Mice were monitored daily until clinical signs of disease were displayed and then were euthanized. Symptoms appeared at around 5 dpi for all strains excepted for AF3 (9 dpi). Epilepsy is observed only in mice infected by EU2. *n* = 6 for each group. **c** Viral burden in blood and brain was measured by RT-qPCR assay at 6 dpi and indicated by TCID50 equivalent per mL or per gram. **d** Transverse sections of the brain (cerebellum) of USUV-infected mice at 6 dpi showing the presence of viral antigen after pan-*flavivirus* staining (associated with luxol blue). *p < 0.05 and **p < 0.01

To compare and study strain virulence in vivo, neonatal immunocompetent Swiss mice were intraperitoneally (i.p.) infected with different USUV strains at 10^3^ tissue culture infectious dose 50% (TCID50) per mouse. Symptoms and mortality were monitored for 20 days. The EU2 strain exhibited the most virulent phenotype as all mice infected by this strain died 10 days post-infection (dpi), whereas the mortality rates for mice infected by EU5, AF3, EU1, EU3, and AF2 strains were 85%, 80%, 56%, 54%, and 53%, respectively, at 20 dpi, the end of the experiment (Fig. 2a). The mean time-to-death was also different between the strains. EU2- and AF2-infected mice died more quickly (8–9 dpi) versus the EU3-, EU5-, and EU1-infected mice (10, 11, and 13 dpi, respectively), whereas AF3-infected mice had a delayed time-to-death with an average of 16 dpi (Fig. 2b). Among infected mice, lethargy and inactivity, limb weakness, and paralysis of the hind limbs appeared on average between 4 and 7 dpi for all strains, except for AF3 for which symptoms appeared later (Fig. 2b). The PBS-inoculated control groups exhibited no signs of disease throughout the experiment. It is interesting to note the induction of convulsive seizures in mice infected with the EU2 strain. These were observed at times of behavioral changes after induction of stress (such as cage changing) and were associated with the epileptiform activities of freezing, tremor, and tail erection. These convulsive seizures were not observed following infection by the other strains, even at later times.

We next investigated the viral burden in the blood and brain of infected mice. In the blood, the viral copy number was higher at 6 dpi in EU2- and EU5-infected mice (*p* < 0.5) (Fig. 2c). All strains tested were neuroinvasive given that we detected the virus in the brains of mice infected with all lineages. However, brain viral burden at 6 dpi was lower in EU1-, EU3-, EU5-, AF2-, and AF3-infected mice compared to EU2-infected mice (*p* < 0.01) (Fig. 2c). EU2-infected brains also showed the presence of more viral antigen by immunohistochemistry staining compared to the other strains, such as AF3- or EU5-infected mice, presenting less viral antigen (Fig. 2d).

Overall, our observations suggest that despite the fact that all strains appear neurotropic and induce neurological impairment in mice, they also have different virulence profiles, particularly the EU2 isolate which appears more pathogenic in this mouse model.

### USUV strains differentially induce brain inflammation

**Fig. 3:**
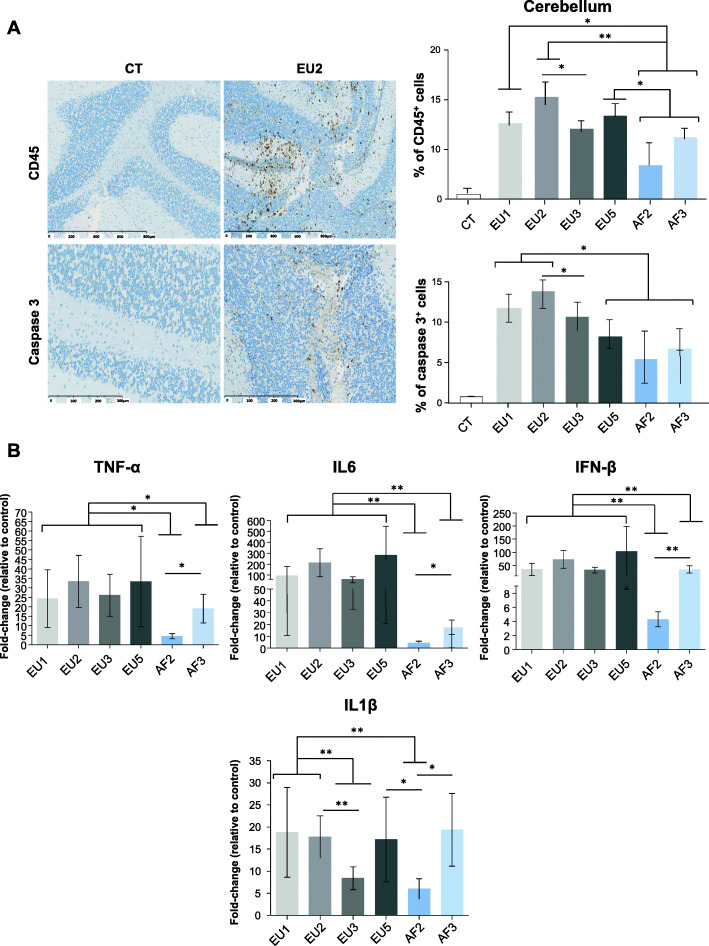
USUV isolates differentially induce cellular infiltration, apoptosis, and inflammation in the mice brain. **a** Left panel: Immunohistochemical CD45 staining (associated with luxol blue) showing inflammatory infiltrates in the infected brain (brown staining) at 6 dpi. Some cells present caspase 3 staining after immunohistochemistry. Right panel: Quantification of CD45-positive cells and caspase 3 positive cells in USUV-infected brain compared to CT. **b** qRT-PCR analysis of TNFα, IL6, IFNβ, and IL1β mRNA from the brain collected at 6 dpi. Each histogram represents the mean ± SEM from 6 independent mice normalized to CT. *p < 0.05 and **p < 0.01

As suggested by previous studies [15, 45, 55] and clinical signs observed in our infected mice in this study (such as epilepsy, hind-limb paralysis, limb weakness, and lethargy), USUV infects, replicates, and damages the brain, in turn causing functional abnormalities. To better describe the brain infection profile following infection by the different isolates, we performed immunohistochemistry in infected brains at 6 dpi. Brain sections were stained with an anti-CD45 antibody, which labels all lymphoid cells. We observed USUV-induced recruitment of CD45^+^ cells for all isolates (Fig. 3a). Cellular infiltration was higher in EU1-, EU2-, and EU5-infected mice compared to the other groups. Caspase-3 staining showed minimal apoptosis, but still more cell death was observed in EU1- and EU2-infected mice (Fig. 3a). To evaluate whether the recruitment of inflammatory cells was correlated with the overexpression of inflammatory cytokines, we analyzed the inflammatory profiles of infected brains at 6 dpi by RT-qPCR (Fig. 3b). We chose markers that have already been associated with viral encephalitis and neurodegenerative diseases, including tumor necrosis factor alpha (TNFα), interleukin 6 (IL6), and interleukin 1 β (IL1β). We detected a strong increase in expression of *Tnfα*, *Il6*, and *Il1β*, but also interferon beta (*Infβ*) in all mouse USUV-infected brains. Increases in expression were generally smaller for African isolates compared to European isolates, except for *Il1β* expression with varying expressions between strains.

### USUV isolates differentially infect and replicate in primary human astrocytes and induce deleterious effects on cellular proliferation and apoptosis

**Fig. 4:**
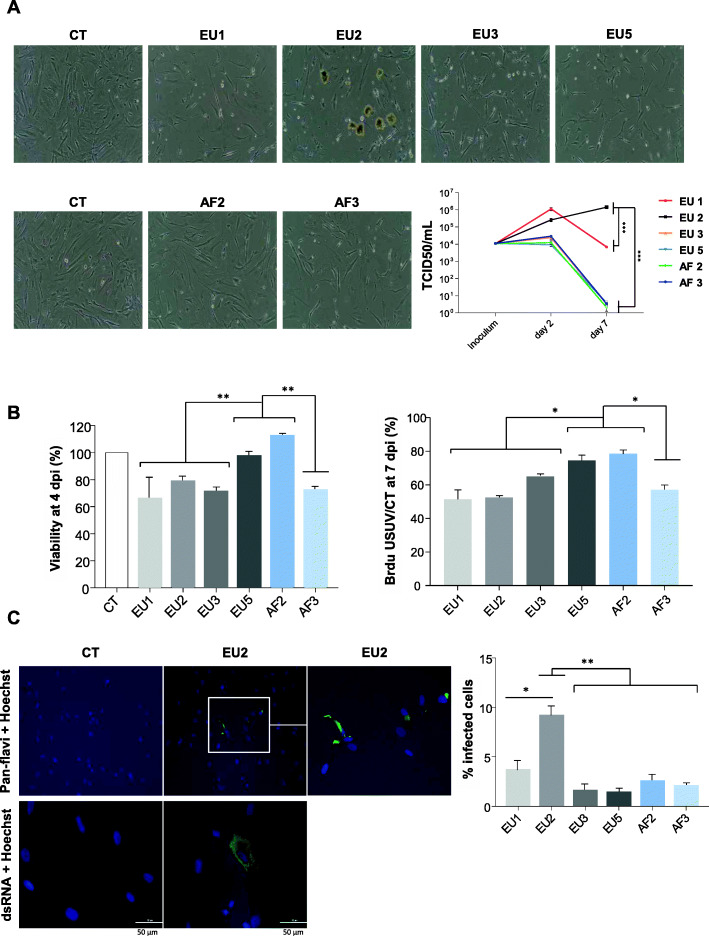
Comparative virulence between USUV strains in primary human astrocytes. Primary human astrocytes were infected with the six lineages of USUV at a MOI of 0.1. **a** Left panel. Bright light images of CT- and USUV-infected astrocytes showing at 7 dpi an atypical CPE which appeared specifically with the EU2 strain. This CPE is characterized by dark clustered cells that detach from the cell mat. Right panel: Supernatants from infected cells were collected at 2 and 7 dpi and subjected to TCID50 measurement on Vero cells. EU2 strain replicates longer in astrocytes. Results are expressed as mean ± SEM of 3 independent experiments. **b** Apoptosis (4 dpi) and cellular proliferation (7 dpi) measured by MTT and BrDU are affected by USUV infection. **c** Left panel. Mock and EU2 USUV-infected cells were fixed at 2 dpi and labeled with the pan-*flavivirus* antibody (in green) or double-stranded RNA antibody (in green) by indirect immunofluorescence. Nuclei are labeled with Hoechst (in blue) immunofluorescence (× 20 and × 40). The white square represents a zoom on infected cells. Scale bar = 50 μm. Right panel: Quantification of infected cells at 2 dpi (using pan-*flavivirus* antibody) showing more infected cells with the EU2 strain. For all experiments, *n* = 3 independent triplicates. *p < 0.05 and **p < 0.01

To better understand the capacity of USUV to infect the brain, we compared USUV isolate virulence in various cell types of the CNS. Firstly, we infected primary human astrocytes with all isolates at a MOI of 0.1. Surprisingly, an atypical CPE was observed at 7 dpi following infection by the EU2 isolate (Fig. 4a). USUV-induced CPE are often characterized by cells rounding up during necrosis, apoptosis, or pyroptosis [56]. Here, CPE induced by EU2 was characterized by dark clusters of cells that detached from the cell mat whereas the other isolates demonstrated typical CPE. In order to determine whether this CPE was characteristic of the EU2 lineage, we analyzed another EU2 isolate (TE18982). We observed the same atypical CPE, suggesting that this observation could be lineage-dependent rather than strain-dependent (Supplemental Figure 1A).

Since the replicative capacity of a virus is considered to be a marker for virulence, we studied the replicative capacity of the different USUV strains in astrocytes. This approach is based on the fact that the most virulent viral strains produce more progeny, which can lead to higher viral densities and therefore greater levels of virulence. Astrocytes infected with the EU1 and EU2 strains released more infectious viral particles at 2 dpi than astrocytes infected with the other strains for which no significant amplification is observed (Fig. 4a). At later times of infection (until 7 dpi), EU2 produced higher titers than the other strains as the production of viral particles further increased while decreasing for all the other strains (Fig. 4a). A similar trend was observed for the other EU2 strain (Supplemental Figure 1B). This is a surprising result because even with CPE the production of viral particles continued to increase over time, suggesting that the virus is persistent and the remaining cells produce a significant amount of infectious viral particles.

To measure the viability of infected astrocytes we performed MTT (3-(4,5-dimethylthiazol-2-yl)-2,5-diphenyltetrazolium bromide) tests at 4 dpi. Results showed more cell death following infection by EU1, EU2, EU3, and AF3 strains compared to EU5 and AF2 strains (Fig. 4b). Given bright-field microscope images of USUV-infected astrocytes seemed to indicate a defect in cellular proliferation, we next explored whether cell division was affected by USUV infection. Astrocytes infected or not by USUV strains were incubated in the presence of the nucleotide analog BrdU and then quantified at 7 dpi by ELISA (Fig. 4b). Compared to non-infected cells, all USUV-infected astrocytes showed a decrease in proliferation at 7 dpi which is however less important for EU5 and AF2 isolates. Pan-*flavivirus* immunofluorescence staining showed very few positive cells but we clearly observed more infected cells after EU2 infection (Fig. 4c). The pattern of antigen staining was characteristic of antigen distribution in the endoplasmic reticulum, a classical site for flavivirus replication (Fig. 4c). Double-stranded RNA staining also indicated an efficient EU2 replication in astrocytes (Fig. 4c). Altogether, these results suggest that the EU2 strain targets primary human astrocytes more efficiently than the other USUV lineages and replicates for longer time.

### Variation of chemokine and cytokine secretion, and gene expression levels in primary human astrocytes between different USUV isolates

**Fig. 5:**
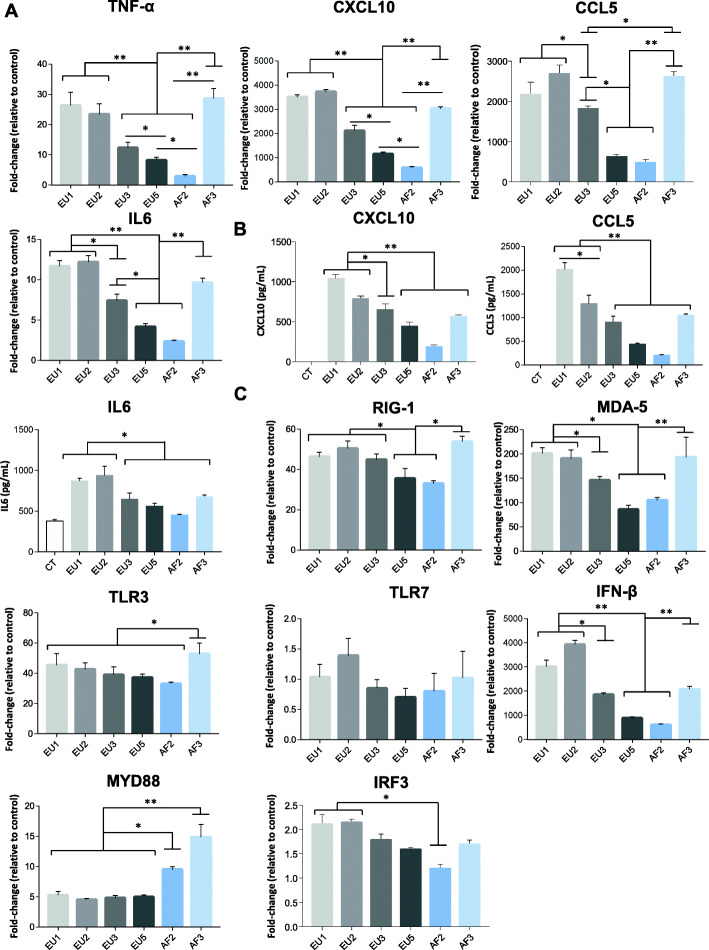
Infection of astrocytes by USUV strains leads to different profiles in the secretion and expression of pro-inflammatory cytokines. **a** qRT-PCR analysis of TNFα, CXCL10, CCL5, and IL6 mRNA collected at 2 dpi from human astrocytes cells infected or not by USUV. Results are expressed as means of the fold regulation. **b** ELISA analyses of CXCL10, CCL5, and IL6 (pg/mL) at 2 dpi. Each histogram represents the mean ± SEM from 3 independent experiments. **c** qRT-PCR analysis of RIG-1, MDA-5, TLR3, TLR7, IFNβ, MYD88, and IRF3 mRNA collected at 2 dpi from human infected astrocytes. Results are expressed as means of the fold regulation normalized to CT (3 independent triplicates). *p < 0.05 and **p < 0.01

Astrocytes, along with microglia, are the predominant source of cytokines and chemokines of CNS resident cells, and thus may act as important processors of neuroinflammation and neurodegeneration [57]. Astrocytes are key players in the inflammatory response during neural infections caused by flavivirus [58, 59]. Consequently, we investigated the mechanisms of induction of the inflammatory signaling elicited by the different USUV isolates in these cells. We first analyzed the modulation of expression of several genes involved in pro-inflammatory cytokine release. We collected mRNAs from USUV- or control-infected human astrocytes at 2 dpi and we performed RT-qPCR to quantify gene transcript levels of selected cytokines (*TNFα*, *CXCL10*, *CCL5*, and *IL6*) (Fig. 5a). We are interested in these cytokines because innate immunity is generally required for clearance of viral infection, and when clearance is ineffective, excessive cytokine release can occur. This can be detrimental and associated with adverse effects, such as CNS disorders. Thus, the main risk during neuroinvasive viral infection is the dispersion of the virus in the CNS, causing induction of inflammatory responses and destruction of neuronal cells. At 2 dpi, we observed a strong upregulation of cytokine mRNA in infected human astrocytes, mainly for the EU1, EU2, and AF3 isolates (Fig. 5a). In parallel, we used enzyme-linked immunosorbent assay (ELISA) to investigate the secretion of CXCL10, CCL5, and IL6 chemokines/cytokines into the culture medium of USUV- and control-infected astrocytes (Fig. 5b). The amount of soluble CXCL10, CCL5, and IL6 was increased in the supernatant of infected astrocytes at 2 dpi, but it varies depending on the isolates used for infection similarly to the results obtained by RT-qPCR (Fig. 5b). Increased expression of CCL5 and CXCL10 could enhance recruitment and activation of immune cells and resident neuronal cells at the site of infection, thereby promoting the clearance of free virus as well as of infected cells in the CNS. Indeed, CCL5 has been reported to have both neuroprotective [60] and direct antiviral activities [61]. Moreover, during the inflammatory response, astrocytes may influence the balance between host protection and neurotoxicity. Considering this, we next investigated the antiviral response. We analyzed the effects of USUV strains on the mRNA level of *Ifnβ* and several cellular pattern recognition receptors (PRRs), as well as some effectors involved in their signaling pathway, including interferon regulatory factor 3 (*IRF3*) and myeloid differentiation primary response 88 (*MYD88*). We observed increased expressions of retinoic acid-inducible gene I (*RIG-I*), melanoma differentiation-associated antigen 5 (*MDA5*), and toll-like receptor 3 (*TLR3*), known to be activated by double-stranded viral RNA during viral replication and involved in IFNβ induction [62]. These genes all exhibited USUV strain-dependent expression levels. We did not observe a significant increase in toll-like receptor 7 (*TLR7*) expression (Fig. 5c). In the literature, PRR expression levels generally increase in concert with ascending responses to viral infection, and suchlike increases are considered to reflect pathway activation [62]. Accumulation of these RNA sensors was accompanied by upregulated mRNA expression levels of IFN signaling pathway components, such as *IFNβ* itself, but also *MYD88* and to a lower extent *IRF3* (Fig. 5c).

Altogether, the gene induction profiles were heterogeneous between the strains. These results suggest that USUV isolates differentially affect cytokine/chemokine production and antiviral responses in primary astrocytes.

### USUV strains differentially infect and replicate in microglial cells

**Fig. 6:**
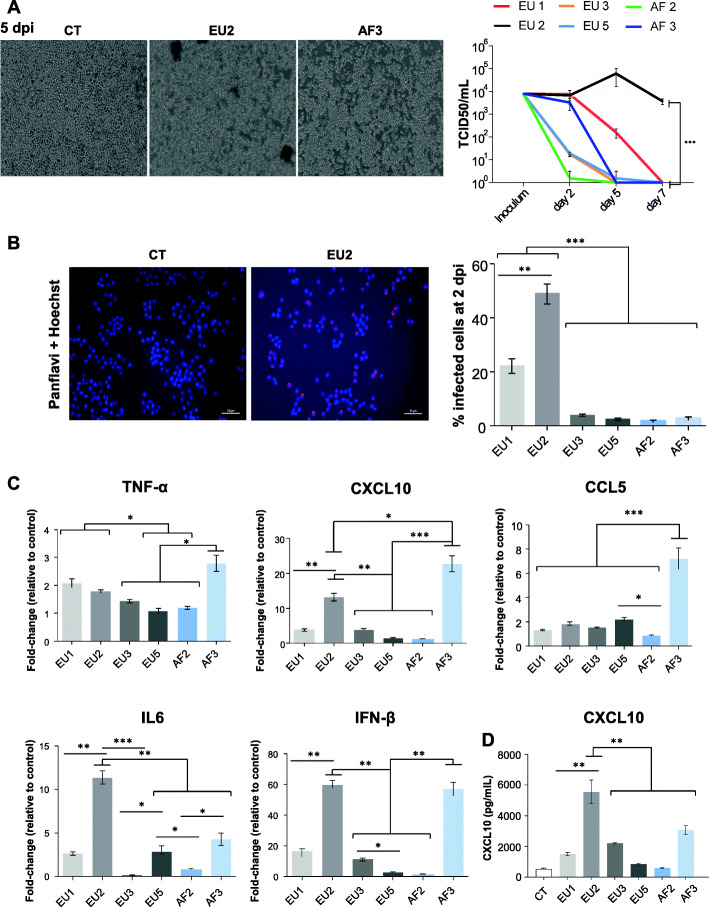
USUV strains replicate differentially in murine microglia and EU2 strains persist longer. Murine microglia were infected with USUV strains at a MOI of 0.1. **a** Left panel: Bright light images of control and USUV-infected microglia at 5 dpi. We observe an atypical CPE- in EU2-infected cells. Right panel: Supernatants from infected cells (MOI 0.1) were collected at 2, 5, and 7 dpi, and subjected to TCID50 measurement. Viral production in USUV-infected microglia shows difference in terms of replication and persistence between strains, with greater virulence for EU2. **b** Left panel: USUV-infected cells were fixed at 2 dpi and labeled with the pan-*flavivirus* antibody (in red) as showed for EU2 strain. Scale bar = 50 μm. The corresponding quantification is indicated on the right panel (*n* = 3 independent experiments). **c** RT-qPCR analysis of TNFα, CXCL10, CCL5, IL6, and IFNβ of mRNA collected at 2 dpi from infected and non-infected (CT) microglial cells. **d** Analyses of CXCL10 by ELISA in the supernatants of CT- or USUV-infected microglia at 2 dpi. Results are expressed as mean ± SEM. *p < 0.05, **p < 0.01, and ***p < 0.001

Microglia represents the resident immune cells of the CNS. During brain infection, they have a critical role in host defense against invading viruses. We previously described that USUV efficiently infects microglia ex vivo in organotypic murine brain slices [45] and the hippocampal area of USUV-infected mice [56]. To determine if USUV isolates can infect microglial cells in vitro, we infected the immortalized murine microglial cell line BV-2 at an MOI of 0.1 [63]. Comparable to our results obtained with astrocytes, we observed an atypical CPE at 5 dpi in microglia infected with the EU2 lineage (Fig. 6a) but not with the other lineages, even at later times (Supplemental Figure 2A). This suggested that this atypical CPE is not a specific feature of infected astrocytes. At 5 dpi, viral titer increase for the EU2 lineage while it decreased for all other isolates (Fig. 6a). Moreover, we detected by pan-*flavivirus* immunofluorescence staining that approximately 50% of cells were EU2-infected, 23% were EU1-infected, and less than 10% were infected by the other strains at 2 dpi (Fig. 6b).

We then investigated the effect of USUV infection on the mRNA expression of key pro-inflammatory cytokines. To compare pro-inflammatory cytokine gene induction between the several isolates in microglia, we collected mRNAs at 2 dpi and performed RT-qPCR to quantify gene transcript levels of *Tnfα*, *Cxcl10*, *Ccl5*, *Il6*, and *Infβ*. We observed a strong upregulation of these cytokine mRNAs, with the exception of *Tnfα*, primarily in EU2- and AF3-infected microglia (Fig. 6c). These data highlight the induction of cytokine signaling by USUV in microglia, particularly by the EU2 and AF3 isolates. This was unexpected given that at 2 dpi the AF3 strain infected a very small percentage of cells (3%, Fig. 6b). On the other hand, the AF3 strain ultimately induced efficient replication and a strong inflammatory response, suggesting that the few infected cells present produced a substantial amount of infectious viral particles. CXCL10 protein secretion measured by ELISA was also significantly increased in microglia infected by both EU2 and AF3 to a lower extent in comparison to the control and other strains (Fig. 6d).

Our results provide evidence that USUV isolates differentially infect and replicate in murine microglial cells. Microglia appear to be more permissive to the EU2 isolate, which also persists longer in this cell type as we already observed in astrocytes.

### All USUV lineages infect pericytes, neurons, and brain endothelial cells, but the EU2 isolate appears more virulent

**Fig. 7:**
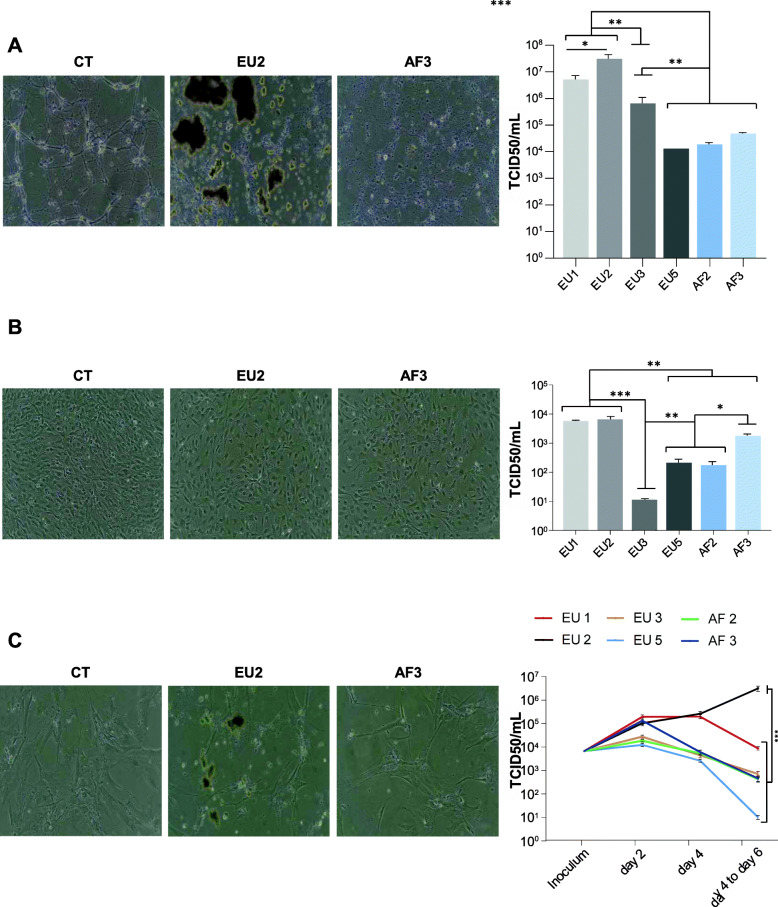
EU2 USUV strain is more virulent and persistent longer in different brain cells. Murine neuronal (**a**), human pericyte (**b**), and human endothelial (**c**) cells cultures were infected with USUV at a MOI of 0.1. Bright light images of CT- and USUV-infected neuron at 4 dpi (**a**) and endothelial cells at 6 dpi (**c**) show atypical CPE in EU2 condition while infection induces no CPE in pericytes at 4 dpi (**b**). Viral quantification for each cell type is expressed in TCID50/mL. Results are expressed as mean ± SEM of 3 independent experiments. *p < 0.05, **p < 0.01, and ***p < 0.001

Next, we compared the replication of the six circulating USUV strains in other CNS cell types: neurons, pericytes, and brain endothelial cells. Brain endothelial cells are highly specialized cells and form the BBB isolating the CNS parenchyma from the blood. The BBB regulates the flow of solutes, cells, and pathogens [64]. Infection and/or disruption of this barrier might be a key event preceding viral invasion. Pericytes are present on the abluminal surface of brain microvessels, and they have a regulatory effect on BBB formation and permeability [65]. Although USUV is currently described as a neurotropic virus and we previously reported that it can effectively replicate in murine neurons, the relative ability of USUV to replicate in cells of the BBB, such as pericytes and brain endothelial cells, is unknown [56]. Similar to our results obtained with astrocytes and microglia, the EU2 strain induced an atypical CPE both in neurons (4 dpi) and in brain endothelial cells (6 dpi) (Fig. 7a, c; Supplemental Figures 2B and 3B). Pericytes on the contrary did not demonstrate any CPE following infection by any of the strains (4 dpi) (Fig. 7b, Supplemental Figure 3A). Moreover, it is noteworthy that neuronal damage was observed at 4 dpi, showing principally neurite destruction in EU1, EU2, EU3, and AF3-infected neurons (Fig. 7a, Supplemental Figure 2B). Productive USUV replication in neurons and pericytes was observed for all strains (Fig. 7a, b). Viral production in murine neurons and in human pericytes at 4 dpi was higher for the European strains, especially the EU2 isolate (Fig. 7a, b). In human brain endothelial cells, we showed that the EU2 strain replicated strongly and was persistent over time (Fig. 7c). Indeed, the viral titer was very comparable between the different lineages at 2 dpi, but at 6 dpi the quantity of EU2 viral particles increased, while it decreased over time from 4 dpi for EU1 and from 2 dpi for the other isolates.

Altogether, our results show that neurons, pericytes, and brain endothelial cells are all permissive to infection by the different USUV strains, but only the EU2 isolate appears again be persistent in these cell types.

### USUV EU2 and AF3 infect and replicate in human brain-like endothelial cells (hBLEC) without perturbation of endothelial integrity

**Fig. 8:**
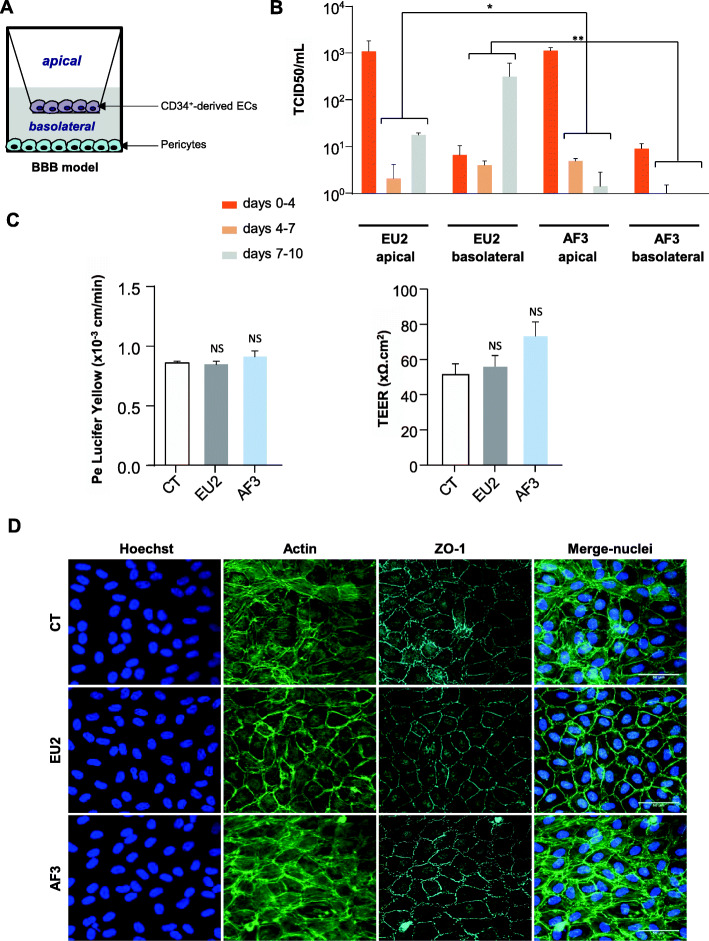
USUV efficiently replicates in a human model of the blood-brain barrier without inducing alteration of endothelium integrity. **a** Human in vitro BBB model composed by CD34^+^ blood cord-derived endothelial cells cultured on transwell filters and placed on top of pericytes. **b** hBLECs were infected with USUV EU2 and AF3 at MOI 0.1. Viral titers from both compartments are indicated at 4, 7, and 10 dpi (*p < 0.05, **p < 0.01). **c** Permeability (Pe) of the Lucifer Yellow (LY) paracellular marker and transendothelial electrical resistance (TEER) was measured at 10 dpi. Each point represents the mean ± SEM of 3 independent experiments. NS = not significant. **d** Immunofluorescence of actin and Zonula occludens (Zo)-1 staining showing no perturbation of endothelium structure (10 dpi). Scale bar = 50 μm

The BBB is very effective in protecting the brain from viruses circulating in the blood. However, neuroinvasive viruses have developed a variety of mechanisms to pass through the BBB and reach the CNS. Several arboviruses, such as WNV and JEV, lead to endothelial integrity impairment and inflammatory molecule production that can disrupt BBB integrity and in turn allow access of the virus to the CNS [66–68]. In this study, we used a human in vitro BBB model which recapitulates the essential characteristics of the endothelial barrier. To acquire BBB characteristics, human CD34^+^ cord blood-derived hematopoietic stem cells were cultivated on culture inserts, in co-culture with pericytes for 6–7 days (Fig. 8a). These hBLEC can form tight junctions and express specific transporters [48] which can allow the study of drug or cell passage through the BBB [69, 70]. We have previously shown that USUV AF2 can replicate in hBLEC and cross the BBB without barrier breakdown [45]. Since replication in brain endothelial cells is sufficient to allow viral transport through BBB, we investigated whether both EU2 (particularly virulent) and AF3 (less virulent) strains could infect and disrupt integrity of the BBB in vitro. Following, hBLEC were infected by USUV on the apical side at an MOI of 0.1. Supernatants were collected at 4, 7, and 10 dpi from the apical (equivalent to blood vessel lumens side) and basolateral (CNS parenchymal side) compartments, and cells were fixed to analyze localization of tight junction proteins, regulating BBB integrity. Thus, endothelial permeability coefficient (Pe) consisting to evaluate the clearance principle of Lucifer Yellow (LY) and transendothelial electrical resistance (TEER) were also measured. Figure 8b shows viral titers in apical and basolateral compartments at 4 dpi (corresponding to the production between 0 and 4 days), 7 dpi (production between 4 and 7 days), and 10 dpi (production between 7 and 10 days). For the EU2 strain, weak viral production was detected at 4 and 7 dpi in the basolateral compartment, but production then increased at 10 dpi. On the other hand, in the apical compartment, the viral production was high at 4 dpi and decreased at 7 dpi, and then increased again at 10 dpi (Fig. 8b). These results suggested an overall efficient production. The AF3 isolate presented a different profile, with no efficient production at 4, 7, or 10 dpi, in both the apical and basolateral compartments (Fig. 8b). Pe of LY appeared not significantly altered by either EU2 or AF3 strains at 10 dpi, both control and infected endothelia displayed permeability coefficients of ~ 0.8 × 10^−3^ cm/min, consistent with a “tight” BBB endothelium (inferior to 1.0) (Fig. 7c). We observed no significant variation in TEER values after infection (Fig. 8c) and the endothelium remained impermeable with no manifestation of architectural disturbance (Fig. 8d).

Our results suggest that despite USUV EU2 being the most virulent strain among the strains tested in this study, EU2 infects the BBB from the luminal side without deleterious effects on BBB integrity.

### Genome analysis highlights mutation in NS5 EU2 strains potentially correlated with virulence

**Fig. 9:**
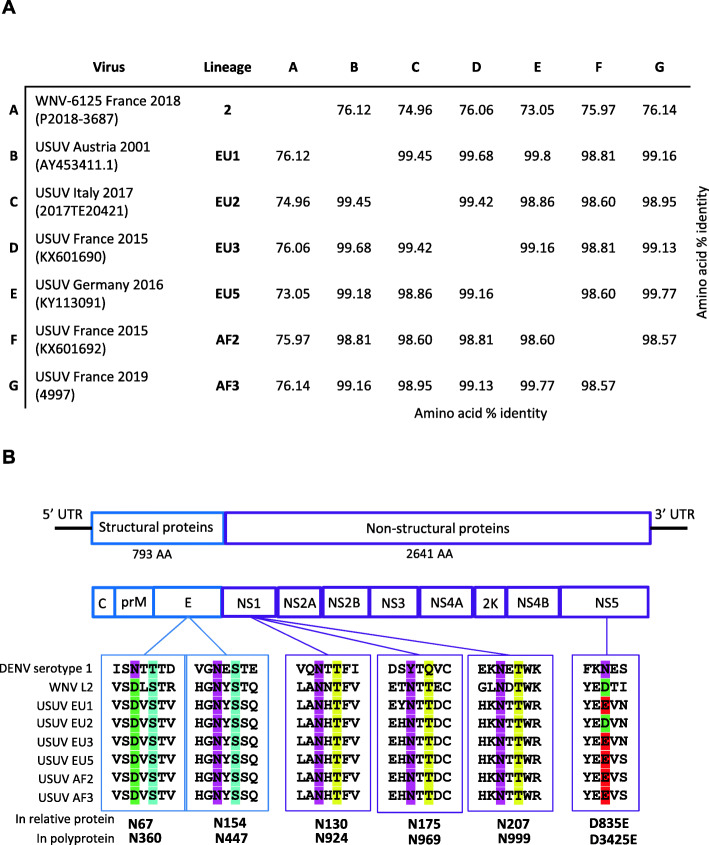
Genetic diversity of USUV strains. **a** Amino acid % identity of the viral polyprotein (MUSCLE, EMBL-EBI). Viral strains and associated lineage are indicated in the table. **b** Annotated viral genome and subsequent viral proteins are shown. A zoom on specific known virulence motifs is presented with amino acid position in the relative viral protein (up) or in the polyprotein (down). Each amino acid is highlighted with a specific color

By comparing the differences at protein level, we found that among USUV strains, the amino acid average pairwise identity was between 98.57 and 99.77% (Fig. 9a). Mutations predicted to influence virulence, pathogenicity, or neuroinvasiveness were then analyzed. USUV strains were compared to a WNV lineage 2 strain which circulated in Europe in 2018 (WNV L2): WNV-6125 France 2018 (P2018-3687). Flaviviruses are described to have N-linked glycosylation sites on their envelope proteins. Glycosylated E protein interacts with cell surface lectins, facilitating attachment and infectivity [50]. As expected, USUV strains have not N-linked glycosylation site at position N67 known to be unique to DENV whereas in all strains we observed glycosylation at N154 (motif NYS) that is highly conserved among flaviviruses. All strains exhibited also the potentially more virulent proline at position NS1-250 [71]. As shown in Fig. 9b, the three potential N-linked glycosylation sites at positions 130, 175, and 207 within the NS1 protein, all molecular determinants of neuroinvasiveness, could be identified in all strains as well. Concerning NS5, we observed, specifically in USUV EU2, an amino acid substitution at position 3425 of the polyprotein, which corresponds to the residue position 835 of the NS5 protein (E3425D/E835D-NS5) (Fig. 9b). We also found this mutation in the second EU2 strain that we used in our study (data not shown). In addition, by analyzing other EU2 sequences available online, we found this mutation in 14/15 isolates (except for JF 266698). This suggests that this substitution could be a characteristic mutation of EU2 lineage. Interestingly, this mutation was previously described for an EU2 strain isolated from a patient with neuroinvasive disease [21]. More precisely, this mutation is localized in the RNA-dependent RNA polymerase domain of the NS5 protein that codes for the viral RNA polymerase. We also identified other unique mutations for EU2 strain (NS1: E36G, R57X, L85, NS2A: 108 T, NS4B: 133S, K349R, T325X, NS5: 55I).

## Discussion

USUV is classified into eight distinct lineages, which can circulate simultaneously in certain areas, especially in Europe. USUV has been repeatedly introduced into Europe from Africa over the last 50 years [72]. Our observations suggest that different isolates from the 6 main USUV lineages can efficiently infect suckling immunocompetent Swiss mice and they all have the ability to access and replicate in the brain parenchyma. However, we reported different virulence profiles between the strains. In particular, the EU2 isolate appeared more pathogenic, whereas the AF3 strain had a significant delay in appearance of symptoms and mortality. One of our striking results is that the EU2 isolate, unlike the other isolates, induced symptoms similar of epileptic seizure including freezing, tremor, and tail erection. Seizures are commonly reported consequences of infection by several neurotropic viruses that can induce neuroinflammatory responses, leading to enhanced neuronal excitability and ultimately contributing to epileptogenesis. For example, another flavivirus ZIKV is known for its induction of epileptic seizures in neonatal infected mice [73]. Exacerbated inflammation could be a consequence as well as a cause of epilepsy, and several inflammatory mediators, such as IL1β, TNF, and IL6, have been detected in surgically resected brain tissue from epileptic patients [74]. The major role of brain inflammation in the development of seizures has also been demonstrated in several models of infectious diseases [75]. However, given the fairly similar inflammatory profiles between the different European isolates in infected mice, exacerbated inflammation does not seem to be the only cause of the convulsive seizures observed following EU2 strain infection.

Viral infections of the CNS are dependent upon viral replication characteristics as well as immune responses to the virus. Once in the brain, neurotropic arboviruses can infect a variety of cells and lead to general neuroinflammation and eventually BBB impairment if cells of the neurovascular unit (NVU) as pericytes, or endothelial cells are targeted, as already described for JEV [76, 77] or DENV [78]. The concept of the NVU was defined by Harder [79] as a structure formed by neurons, astrocytes, pericytes, microglia, oligodendrocytes, and endothelial cells. Interactions occur between neural, glial, and vascular components of the NVU in response to physiological stimuli or neuroimmune responses. Overall, these interactions maintain CNS homeostasis. NVU cells are direct or indirect targets of neurotropic flaviviruses. Infection of these cells can induce the release of soluble mediators that are involved in neuroinflammation and, in some cases, disruption of the blood-brain barrier (BBB). However, CNS resident cells have developed innate antiviral immune strategies to defend against neurotropic viruses. If neurons are a major target, other cell types such as astrocytes, microglia, pericytes, and endothelial cells can also become infected and participate to the resolution of infection by generating immune responses. Microglia and astrocytes are the main cells in the CNS responsible for initiating, regulating, and maintaining neuroimmune responses to viral infections. Activated glial cells are known to produce numerous mediators, including cytokines and chemokines, that orchestrate both the defense against and the pathogenicity of CNS viral infections [80]. Astrocytes can be infected by different flaviviruses, including ZIKV, WNV, JEV, or tick-borne encephalitis virus (TBEV) [59, 81, 82], as well as USUV [56]. Interestingly, as observed in suckling immunocompetent Swiss mice, the EU2 USUV isolate also resulted in higher viral infection/replication in a large panel of neuronal cells. In this regard, EU2 infection resulted in higher susceptibility and proportion of infected cells in astrocytes, where we observed increased virulence with two different strains, suggesting that the effect is potentially lineage-dependent rather than strain-dependent. The ability of the USUV strains to differentially replicate in NVU cells may favor the capacity of the virus to undergo a second round of infection into the CNS. Thus, NVU cells may play a central role not only in the initial dissemination of different USUV strains to the CNS, but also in secondary waves of spread, both likely to exacerbate USUV-mediated neuropathology.

CNS cells express a number of PRRs and are able to respond to pathogen-associated molecular patterns (PAMPs; derived from pathogens) and to damage-associated molecular patterns (DAMPs; released upon CNS damage). USUV appears to induce a different cytokine response depending on the cell type and the strain. For example, the EU2 isolate replicated more efficiently and induced stronger cytokine responses in microglia, one of the main effector cells in neuroinflammation. The AF3 strain had a similar profile; nevertheless, it replicated for a shorter time in these cells. The fact that the EU2 strain replicates more and for a longer time compared to the other strains in several CNS cell types could explain the strong deleterious effect of the infection observed in vivo. The host innate antiviral response plays a central role in controlling viral replication. Following USUV infection, the inflammatory context is associated with an important antiviral response, as observed by upregulation of IFNβ in the infected brain and in neuronal cells. In infected astrocytes, there was increased expression of genes involved in the antiviral response, such as IFNβ and IRF3, mainly for EU1 and EU2 strain infections. Nonetheless, replication was also highest for these two strains. These results suggest that the IRF3-dependent arm of the host antiviral response does not differentially restrict USUV strain replication within infected cells. Moreover, the host antiviral response appears to play a minimal role in the differential susceptibility of astrocytes to different USUV strains. The absence of an efficient protective effect of type 1 IFNs has been demonstrated for numerous other neurotropic flaviviruses, such as DENV and WNV [83]. Previous studies have shown the induction of antiviral responses despite USUV replication, suggesting that USUV does not harbor mechanisms that efficiently interfere with IFN induction [56]. The mechanisms used by USUV to interfere with this IFN response in order to establish a productive infection are currently not known. RIG-I, TLR3, and MDA5 are well-conserved cytoplasmic PRRs that detect viral RNAs during infection and activate the type I IFN-mediated antiviral immune response. RNA levels of PRRs generally increase in concert with viral infection and are considered to reflect pathway activation. While RIG-I or TLR3 were overexpressed following USUV infection and this did not vary strongly between different strains, MDA5 overexpression was greater in isolates with strong IFNβ induction, including the EU1, EU2, and AF3 strains.

Host innate responses leading to pro-inflammatory cytokine and chemokine production are thought to modulate BBB integrity and enable viruses to access the brain [84]. The brain endothelium constitutes a primary barrier to virus neuroinvasion. Using an in vitro BBB model that exhibits the physiological characteristics of the brain endothelium [48], we demonstrated that the EU2 isolate, the most virulent strain we tested, can infect and replicate in hBLEC. Here, viral particles were released in the basolateral compartment (which would correspond to the parenchymal side) for a prolonged time (unlike the AF3 strain), potentially increasing the capacity of the EU2 strain to cross the BBB. However, this isolate did not disturb endothelial integrity. It is generally suggested that for viral invasion BBB disruption is a pivotal event. Yet, numerous studies show that neurotropic flaviviruses can reach the CNS by crossing the BBB without breaking the barrier, allowing free virus to spread in the CNS. Our findings are consistent with reports that the capacity of virus to cross the BBB to reach the CNS is not systematically the determining factor for neuropathogenicity. Increasing evidence suggests that viral entry into the CNS is often a multistep process that can occur through one of several routes. Some neurotropic viruses can directly infect the endothelial cells of the brain and release viral particles into the parenchyma. Viral neuroinvasion can thus occur via replication of the virus within NVU cells prior to the breakdown of the BBB. This mechanism is thought to be shared by neuroinvasive viruses, including the closely related flavivirus WNV, and enhances dissemination within the CNS [85]. Our results suggest that the differential invasion of the brain by the several different USUV strains does not seem to be linked to strain-dependent BBB disturbance, but rather to a different capacity to infect and replicate in the cells forming the BBB.

Another interesting observation is the presence of a specific and atypical CPE following EU2 lineage infection in all CNS components (with the exception of pericytes). The characteristics of CPE caused by flaviviruses vary according to the host cell and involved various factors, including viral receptors, host genetics, defective viral particles, and immune response [86]. CPE induced by USUV is usually characterized by a diffuse CPE corresponding to cell rounding, shrinkage, and lysis. The atypical CPE we observed with two different isolates of EU2 was characterized by the presence of dead cells floating in clusters and could potentially be correlated with the higher virulence of this lineage. We could expect a relation between viral infectivity and virulence, CPE thus being unique to specific USUV lineages. Cellular and/or viral factors involved in this specific CPE induced by EU2 strains remain to be determined.

Virulence is an adaptive process and the result of interactions between numerous factors, involving both viral and host cell factors. These could include for example tropism, induction of neuronal apoptosis, resistance to interferon, regulation of cytokines and chemokines, induction of matrix metalloproteinase, quasi-species generation, or upregulation of major histocompatibility complex class I expression. It is therefore clear that virulence is a multifactorial process and many aspects need to be studied in order to elucidate viral pathogenicity. For example, the neuroinvasive potential of WNV is strain-dependent, but mainly due to differential strain ability to reach the CNS [43]. In some cases, this difference could also be due to different replication capacities in BBB cells or directly in neurons, as already demonstrated for WNV [59] and Semliki Forest Virus [87]. It is documented that there is a close relationship between European USUV strains (nucleotide identity 99%) while there is a greater divergence between African USUV isolates (nucleotide identity 96%). Several structural and nonstructural genes (prM, E, NS1, NS3, and NS5) have exhibited greater nucleotide than amino acid divergence [72]. The virulence of flaviviruses has often been associated with E protein glycosylation [50] and glycosylation of the NS1 protein [88]. One possible viral factor of virulence is the presence of specific N-linked glycans on the E protein, correlating thus with the ability to invade the CNS [43, 50, 89]. All strains analyzed exhibited classical markers of neurovirulence previously described, which is consistent with the fact that all of our strains are able to invade the brain. Nevertheless, we identified specific mutations in EU2 strain (NS1: E36G, R57X, L85, NS2A: 108 T, NS4B: 133S, K349R, T325X, NS5: 55I, E835D). The discovery of amino acid substitution in the RdRp domain of the NS5 protein (E3425D/E835D-NS5) in both EU2 strains is particularly interesting, as this substitution is not seen in the other strains from other lineages. In addition, by analyzing other EU2 sequences available online, we found this mutation in 14/15 isolates (except for JF 266698). This suggests that this substitution could be a characteristic mutation of EU2 lineage. For instance, this aa change was also found in WNV and in other neurovirulent flaviviruses (JEV, Kunjin virus (KUNV), and Murray Valley encephalitis virus (MVEV)) and have been suggested to be related with viral neuroinvasiveness [21]. Furthermore, substitutions in virtually equivalent positions were associated with variation in the capacity of WNV to invade the CNS [21] and an E to D substitution in the NS5 protein of TBEV affects the virulence in mice underlying the contribution of viral RdRp to flavivirus neurovirulence [90]. This mutation was previously described in an EU2 strain isolated from a patient with neuroinvasive disease and was suggested to impact the efficiency of viral replication and virulence which our results seem to corroborate. Indeed, NS5 protein may have an effect on the efficiency of viral replication as its codes for the RNA-dependent RNA polymerase involved in the replication complex [21]. Association of these several mutations with virulence remains to be elucidated. For this, reverse genetic systems will be helpful to allow the identification of the molecular and cellular mechanisms involved in USUV pathogenicity.

There is a strong probability that the strains used in this study were not derived due to extensive passaging (no more than 5 passages for all strains), so altogether our data indicate that there are intrinsic differences in the pathogenicity/virulence of USUV isolates in vivo and in vitro as previously described for other flaviviruses, such as ZIKV and WNV [41, 42]. Recent studies in rodents [91], immunocompetent mice [92], and chicken embryos [93] also demonstrate different results depending on the strain used. Is should be noted that no difference of mortality was reported in chicken embryos infected with several USUV strains [93]. This difference can be related to many factors (such as inoculation sites, number of viral passages, difference in models (birds versus mammals, embryos versus neonatal animals…). For this reason, it is important to investigate whether the differences in virulence observed in our models have clinical relevance. As reported in the literature, neurological complications are mainly described after infection with an EU2 strain, which is also a lineage frequently circulating in the south of Europe. It can consequently be hypothesized that the USUV EU2 lineage, characterized by a higher infection and viral production, longer persistence, and induction of an atypical CPE, could be more pathogenic for humans and/or birds and likely better adapted to the vector hosts.

## Conclusion

In conclusion, our study on the pathogenic properties of USUV strains circulating in Europe contributes to a better understanding of USUV pathogenesis. To define the host and viral factors involved in pathogenesis, the naturally occurring diversity in virulence among viral strains provides a robust model system. Monitoring the global diversity and distribution of circulating USUV strains, as well as characterizing their neurotropism, will aid in the threat assessment and epidemiological modeling if future USUV outbreaks occur.

## Supplementary Information

**Additional file 1: Supplemental Figure 1.** Two USUV EU2 strains induce a specific CPE and persistent viral replication in astrocytes. **Supplemental Figure 2.** Differential CPE in USUV-infected murine microglia and neurons cells. **Supplemental Figure 3.** USUV isolates induces CPE in human endothelial cells with an atypical shape for EU2 while there is no CPE in pericytes.

## Data Availability

All data generated or analyzed during this study are included in this published article and its supplementary information files.

## Abbreviations

AF: Africa
BBB: Blood-brain barrier
BRDU: Bromodeoxyuridine
C: Capsid
CNS: Central nervous system
CPE: Cytopathic effect
DAMPs: Damage-associated molecular patterns
DENV: Dengue virus
Dpi: Days post-infection
E: Envelope
ELISA: Enzyme-linked immunosorbent assay
EU: Europe
hBLEC: Human Brain-like Endothelial Cells
IFN: Interferon
*Ifnar1*^*-/-*^: Lacking the interferon type 1 receptor
IL1β: Interleukin 1 β
IL6: Interleukin 6
i.p.: Intraperitoneally
IRF3: Interferon regulatory factor 3
JEV: Japanese encephalitis virus
KUNV: Kunjin virus
LY: Lucifer Yellow
MDA5: Melanoma differentiation-associated antigen 5
MVEV: Murray Valley encephalitis virus
MTT: 3-(4,5-Dimethylthiazol-2-yl)-2,5-diphenyltetrazolium bromide
MYD88: Myeloid differentiation primary response 88
NVU: Neurovascular unit
ORF: Open reading frame
PAMPs: Pathogen-associated molecular patterns
PFA: Paraformaldehyde
prM/M: Pre-membrane/membrane
PRRs: Pattern recognition receptors
RHEM: Réseau d’Histologie Expérimentale de Montpellier
RIG-I: Retinoic acid-inducible gene I
RT-qPCR: Quantitative reverse transcriptase PCR assay
TBEV: Tick-borne encephalitis virus
TCID50: Tissue culture infectious dose 50%
TEER: Transendothelial electrical resistance
TLR: Toll-like receptor
TNFα: Tumor necrosis factor alpha
USUV: Usutu virus
WNV: West Nile virus
ZIKV: Zika virus
