# Correction to: Differential neurovirulence of Usutu viruslineages in mice and neuronal cells

**DOI:** 10.1186/s12974-021-02109-y

**Published:** 2021-02-23

**Authors:** Marion Clé, Orianne Constant, Jonathan Barthelemy, Caroline Desmetz, Marie France Martin, Lina Lapeyre, Daniel Cadar, Giovanni Savini, Liana Teodori, Federica Monaco, Jonas Schmidt-Chanasit, Juan-Carlos Saiz, Gaëlle Gonzales, Sylvie Lecollinet, Cécile Beck, Fabien Gosselet, Philippe Van de Perre, Vincent Foulongne, Sara Salinas, Yannick Simonin

**Affiliations:** 1 Pathogenesis and Control of Chronic Infections, Université de Montpellier,INSERM, EFS, Montpellier, France; 2 BioCommunication en CardioMétabolique(BC2M), Montpellier University, Montpellier, France; 3 Université deMontpellier, CNRS, Viral Trafficking, Restriction and Innate Signaling, Montpellier, France; 4 Bernhard Nocht Institute for Tropical Medicine, WHO Collaborating Centre for Arbovirus and Haemorrhagic Fever Reference and Research, 20359 Hamburg, Germany; 5 OIE Reference Centre for West NileDisease, Istituto Zooprofilattico Sperimentale“G. Caporale”, 46100 Teramo, Italy; 6 Faculty of Mathematics, Informatics and Natural Sciences, UniversitätHamburg, 20148 Hamburg, Germany; 7 Department of Biotechnology, INIA, Madrid, Spain; 8 UPE, Anses Animal Health Laboratory, UMR1161 Virology, INRA, Anses, ENVA, Maisons-Alfort, France; 9 Blood-Brain Barrier Laboratory (BBB Lab), University of Artois, UR2465, F-62300 Lens, France; 10 Centre Hospitalier Universitaire de Montpellier, Montpellier, France

**Correction to: J Neuroinflammation (2021) 18:11**

**https://doi.org/10.1186/s12974-020-02060-4**

Following publication of the original article [1], the authors noticed that there were error bars offset in Figs. 3, 5 and 6 in the published version of this article. Presented here are the corrected Figs. 3, 5 and 6. The original article has been updated.

**Fig. 3:**
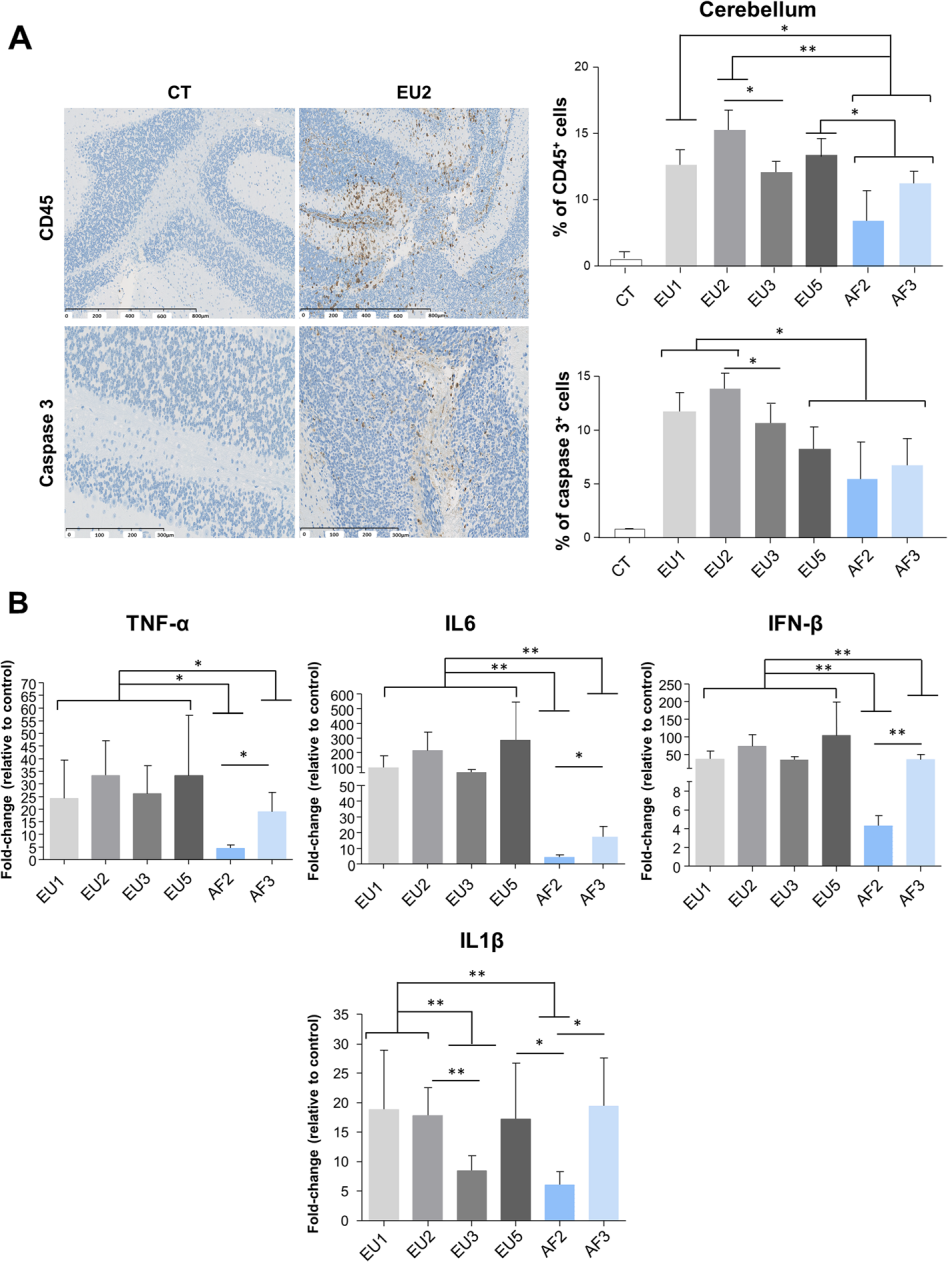
USUV isolates differentially induce cellular infiltration, apoptosis, and inflammation in the mice brain. **a** Left panel: Immunohistochemical CD45 staining (associated with luxol blue) showing inflammatory infiltrates in the infected brain (brown staining) at 6 dpi. Some cells present caspase 3 staining after immunohistochemistry. Right panel: Quantification of CD45-positive cells and caspase 3 positive cells in USUV-infected brain compared to CT. **b** qRT-PCR analysis of TNFα, IL6, IFNβ, and IL1β mRNA from the brain collected at 6 dpi. Each histogram represents the mean ± SEM from 6 independent mice normalized to CT. *p < 0.05 and **p < 0.01

**Fig. 5:**
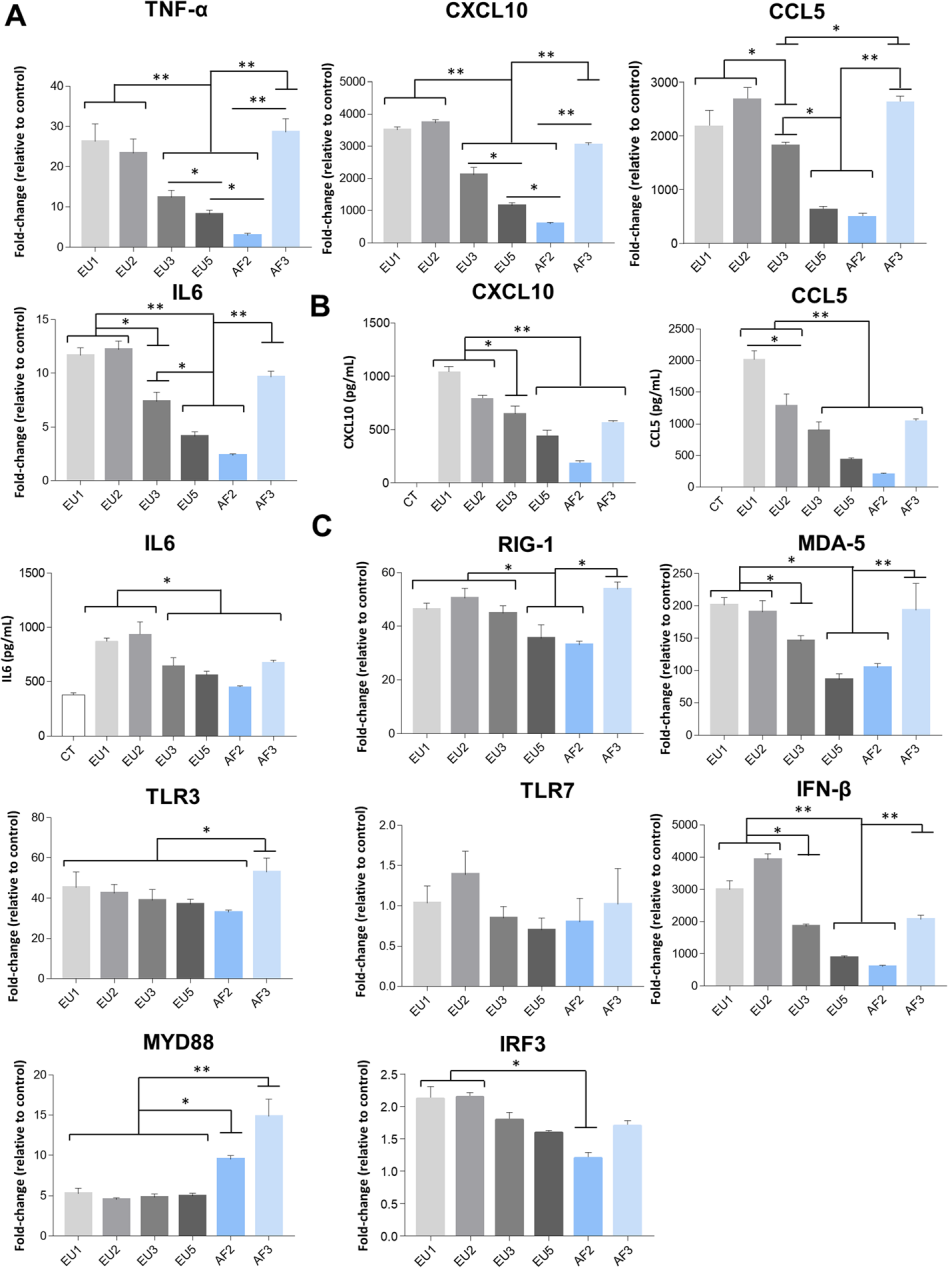
Infection of astrocytes by USUV strains leads to different profiles in the secretion and expression of pro-inflammatory cytokines. **a** qRT-PCR analysis of TNFα, CXCL10, CCL5, and IL6 mRNA collected at 2 dpi from human astrocytes cells infected or not by USUV. Results are expressed as means of the fold regulation. **b** ELISA analyses of CXCL10, CCL5, and IL6 (pg/mL) at 2 dpi. Each histogram represents the mean ± SEM from 3 independent experiments. **c** qRT-PCR analysis of RIG-1, MDA-5, TLR3, TLR7, IFNβ, MYD88, and IRF3 mRNA collected at 2 dpi from human infected astrocytes. Results are expressed as means of the fold regulation normalized to CT (3 independent triplicates). *p < 0.05 and **p < 0.01

**Fig. 6:**
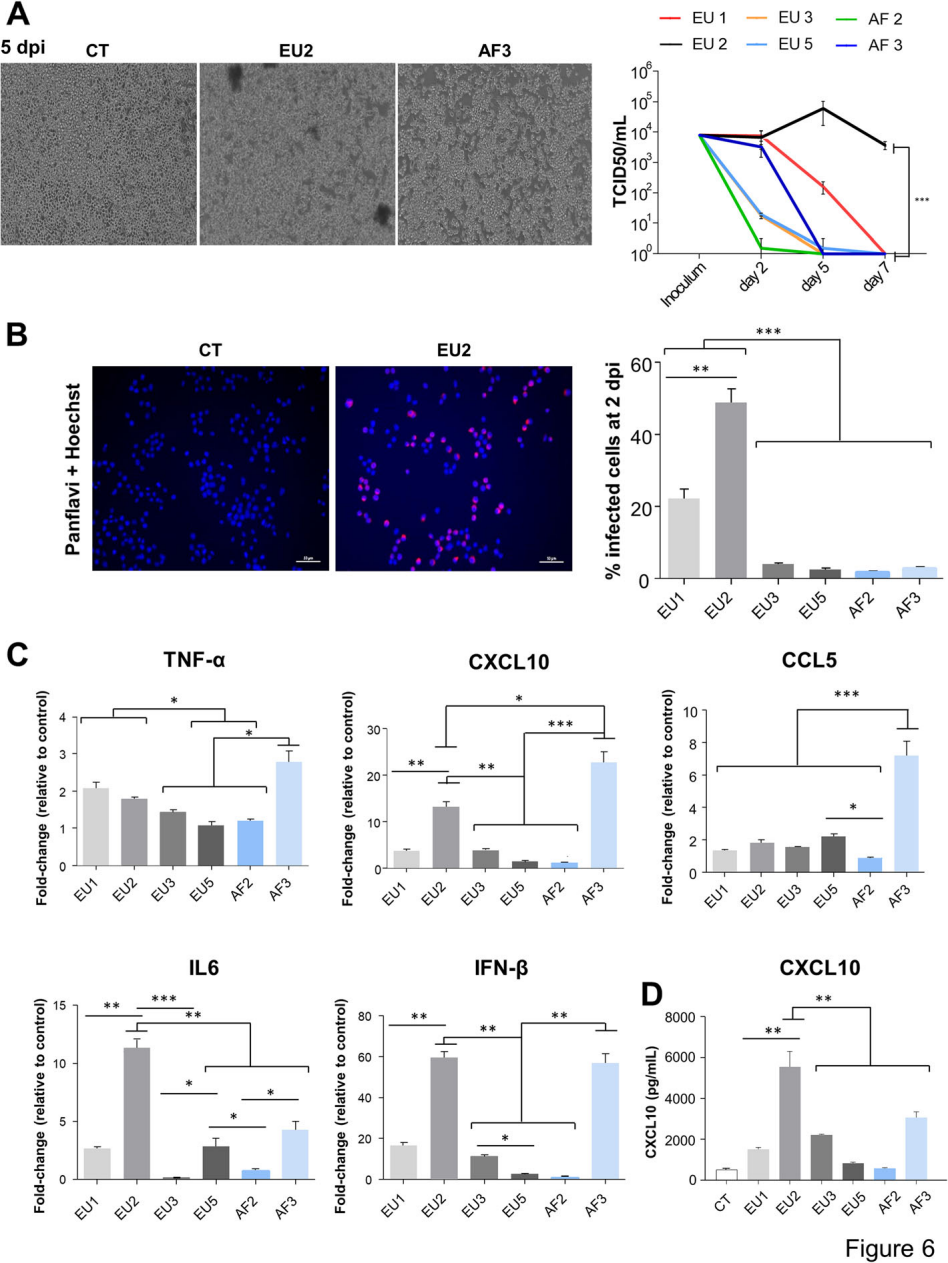
USUV strains replicate differentially in murine microglia and EU2 strains persist longer. Murine microglia were infected with USUV strains at a MOI of 0.1. **a** Left panel: Bright light images of control and USUV-infected microglia at 5 dpi. We observe an atypical CPE- in EU2-infected cells. Right panel: Supernatants from infected cells (MOI 0.1) were collected at 2, 5, and 7 dpi, and subjected to TCID50 measurement. Viral production in USUV-infected microglia shows difference in terms of replication and persistence between strains, with greater virulence for EU2. **b** Left panel: USUV-infected cells were fixed at 2 dpi and labeled with the pan-*flavivirus* antibody (in red) as showed for EU2 strain. Scale bar = 50 μm. The corresponding quantification is indicated on the right panel (*n* = 3 independent experiments). **c** RT-qPCR analysis of TNFα, CXCL10, CCL5, IL6, and IFNβ of mRNA collected at 2 dpi from infected and non-infected (CT) microglial cells. **d** Analyses of CXCL10 by ELISA in the supernatants of CT- or USUV-infected microglia at 2 dpi. Results are expressed as mean ± SEM. *p < 0.05, **p < 0.01, and ***p < 0.001
