# Supplementary material for: Differential neurovirulence of Usutu virus lineages in mice and neuronal cells
Source: J Neuroinflammation. 2021 Jan 6;18:11. doi: 10.1186/s12974-020-02060-4 (PMC7789689; doi:10.1186/s12974-020-02060-4)
Supplement: Supplementary file 1 — Additional file 1: Supplemental Figure 1. Two USUV EU2 strains induce a specific CPE and persistent viral replication in astrocytes. Supplemental Figure 2. Differential CPE in USUV-infected murine microglia and neurons cells. Supplemental Figure 3. USUV isolates induces CPE in human endothelial cells with an atypical shape for EU2 while there is no CPE in pericytes. [file 12974_2020_2060_MOESM1_ESM.pdf]

Supplemental Figures Legends

Supplemental Figure 1. Two USUV EU2 strains induce a specific CPE and persistent viral replication in astrocytes

Human astrocyte cells were infected with two strains from EU2 lineage (20421 and 18982) at a MOI of 0,1. (A) Bright light images of human astrocytes infected at 7 dpi show similar atypical CPE with the two strains. (B) Supernatants from infected astrocyte cells (MOI 0,1) were collected at 2, 7, and 11 dpi and subjected to TCID50 measurement on Vero cells. EU2 strains have similar replication and persistence over time. Results are expressed as mean ± SEM of 3 independent experiment. (C) Cellular proliferation (7 dpi) and apoptosis (4 dpi) cells measured respectively with BrdU and MTT are affected similarly with the two EU2 isolates. NS : Not significant.

Supplemental Figure 2. Differential CPE in USUV-infected murine microglia and neurons cells

(A) Bright light images of infected murine microglial cells at 5 dpi and murine neurons (B) at 4 dpi (MOI of 0,1) showed specific CPE in EU2 USUV-infected condition.

Supplemental Figure 3. USUV isolates induces CPE in human endothelial cells with an atypical shape for EU2 while there is no CPE in pericytes

(A) USUV infection in pericytes (at 4pi) does not induce CPE unlike endothelial cells (B) where an atypical CPE is observed with the EU2 strain (at 6 dpi). (B) USUV infection in pericytes (at 4pi) does not induce CPE unlike endothelial cells (B) where an atypical CPE is observed with the EU2 strain (at 6 dpi).

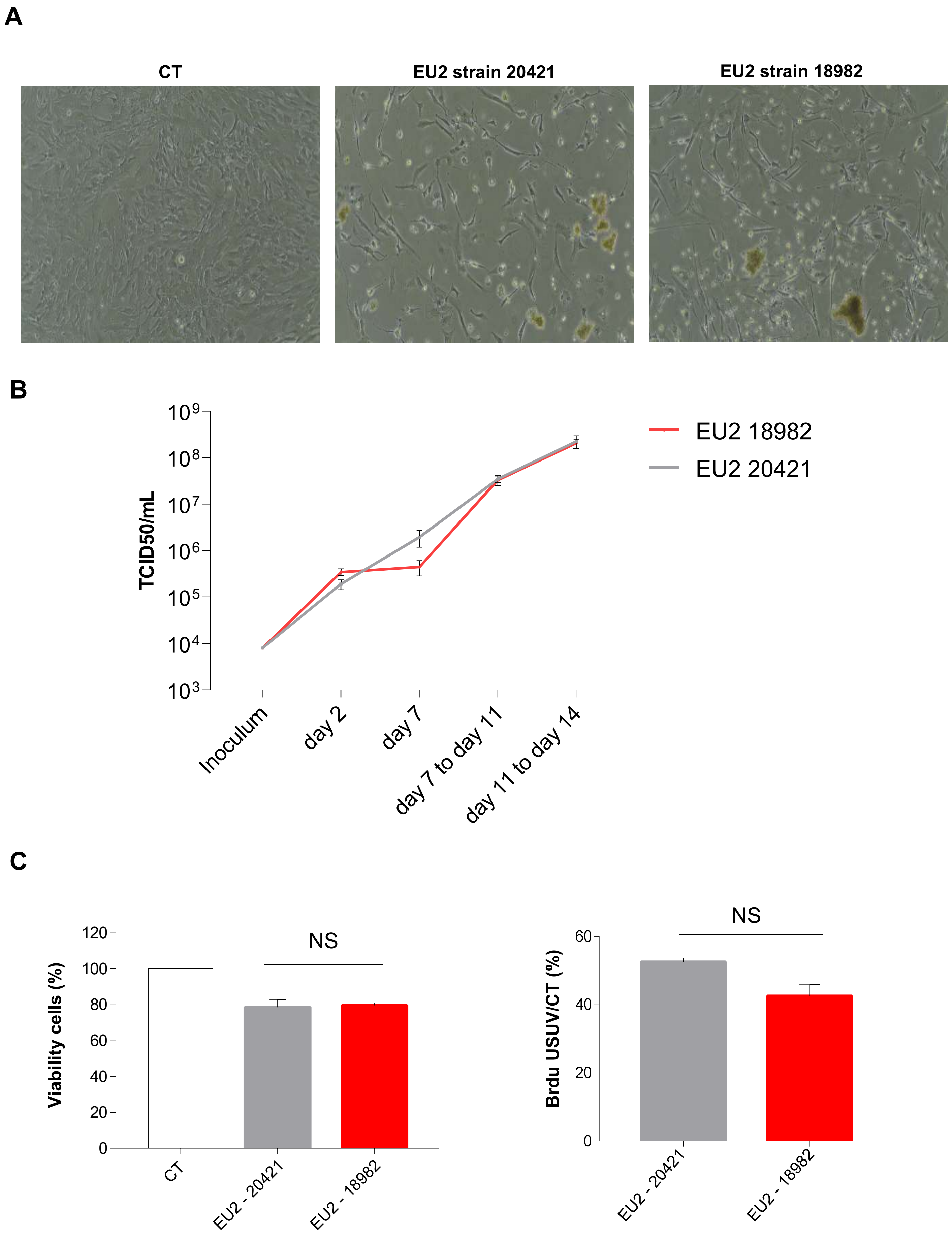

Supp Figure 1

A

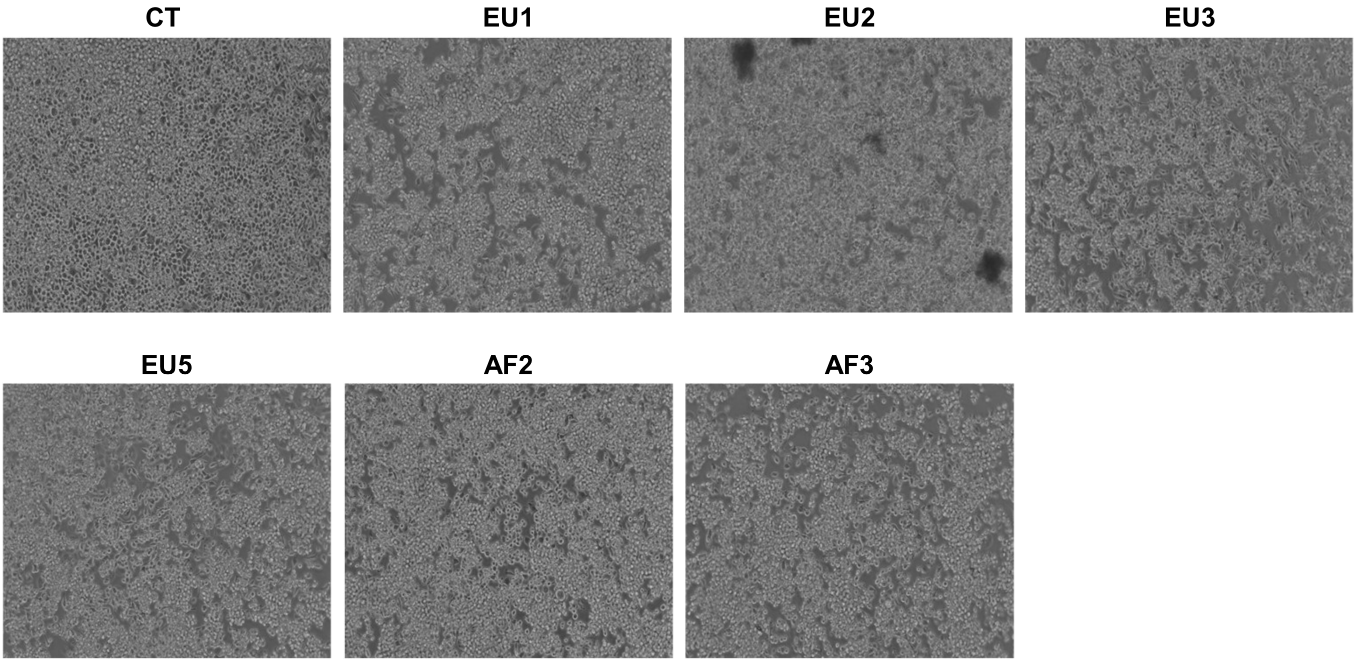

B

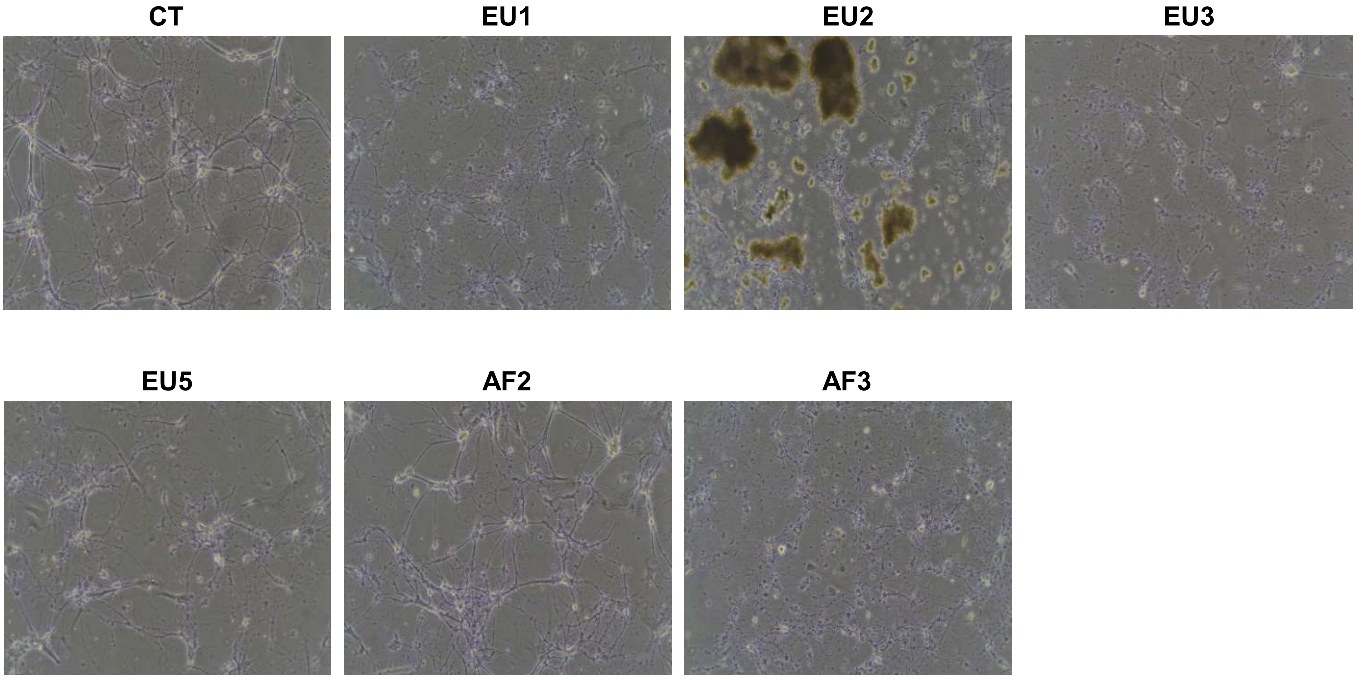

Supp Figure 2

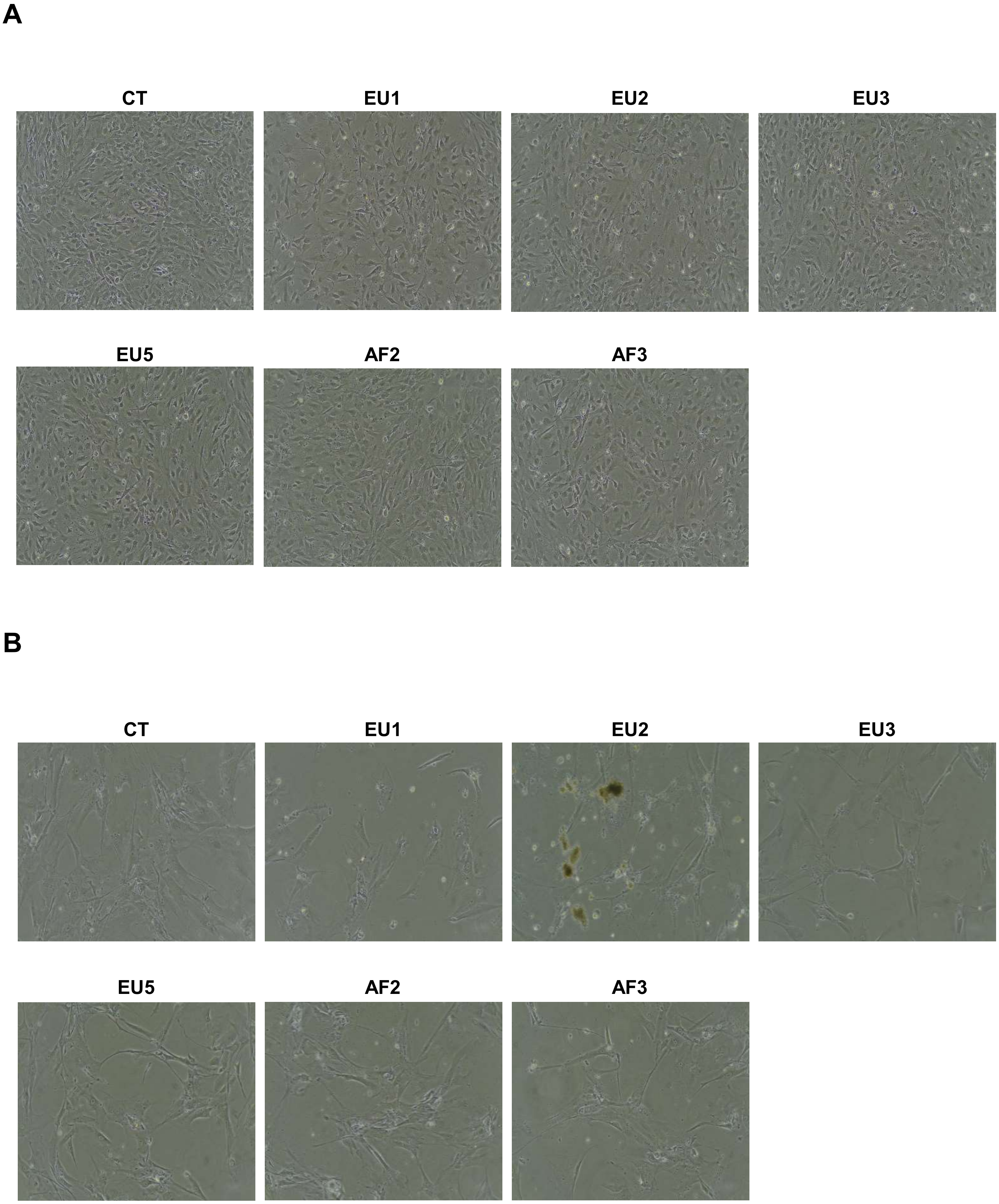

Supp Figure 3
